# Neonatal hyperoxia in mice triggers long-term cognitive deficits via impairments in cerebrovascular function and neurogenesis

**DOI:** 10.1172/JCI146095

**Published:** 2022-11-15

**Authors:** Marissa A. Lithopoulos, Xavier Toussay, Shumei Zhong, Liqun Xu, Shamimunisa B. Mustafa, Julie Ouellette, Moises Freitas-Andrade, Cesar H. Comin, Hayam A. Bassam, Adam N. Baker, Yiren Sun, Michael Wakem, Alvaro G. Moreira, Cynthia L. Blanco, Arul Vadivel, Catherine Tsilfidis, Steven R. Seidner, Ruth S. Slack, Diane C. Lagace, Jing Wang, Baptiste Lacoste, Bernard Thébaud

**Affiliations:** 1 Regenerative Medicine Program, Ottawa Hospital Research Institute, Ottawa, Ontario, Canada.; 2 Department of Cellular and Molecular Medicine, Faculty of Medicine, University of Ottawa, Ottawa, Ontario, Canada.; 3 Neuroscience Program, Ottawa Hospital Research Institute, Ottawa, Ontario, Canada.; 4 University of Texas Health Science Center at San Antonio, San Antonio, Texas, USA.; 5 Department of Computer Science, Federal University of São Carlos, São Carlos, Brazil.; 6 Department of Biochemistry, Microbiology, and Immunology, Faculty of Medicine, University of Ottawa, Ottawa, Ontario, Canada.; 7 Thermo Fisher Scientific, Burlington, Ontario, Canada.; 8 Department of Ophthalmology, Faculty of Medicine, University of Ottawa, Ottawa, Ontario, Canada.; 9 University of Ottawa Brain and Mind Research Institute, Ottawa, Ontario, Canada.; 10 Children’s Hospital of Eastern Ontario Research Institute, Ottawa, Ontario, Canada.

**Keywords:** Neuroscience, Stem cells, Behavior, Neurodevelopment, Neuronal stem cells

## Abstract

Preterm birth is the leading cause of death in children under 5 years of age. Premature infants who receive life-saving oxygen therapy often develop bronchopulmonary dysplasia (BPD), a chronic lung disease. Infants with BPD are at a high risk of abnormal neurodevelopment, including motor and cognitive difficulties. While neural progenitor cells (NPCs) are crucial for proper brain development, it is unclear whether they play a role in BPD-associated neurodevelopmental deficits. Here, we show that hyperoxia-induced experimental BPD in newborn mice led to lifelong impairments in cerebrovascular structure and function as well as impairments in NPC self-renewal and neurogenesis. A neurosphere assay utilizing nonhuman primate preterm baboon NPCs confirmed impairment in NPC function. Moreover, gene expression profiling revealed that genes involved in cell proliferation, angiogenesis, vascular autoregulation, neuronal formation, and neurotransmission were dysregulated following neonatal hyperoxia. These impairments were associated with motor and cognitive decline in aging hyperoxia-exposed mice, reminiscent of deficits observed in patients with BPD. Together, our findings establish a relationship between BPD and abnormal neurodevelopmental outcomes and identify molecular and cellular players of neonatal brain injury that persist throughout adulthood that may be targeted for early intervention to aid this vulnerable patient population.

## Graphical abstract

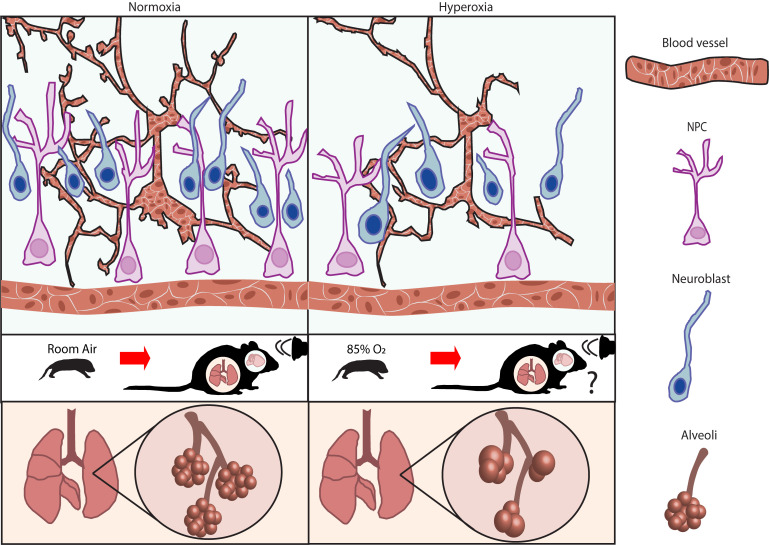

## Introduction

Preterm birth (<37 weeks) is the leading cause of death in children under the age of 5 (1). A chronic lung disease known as bronchopulmonary dysplasia (BPD) represents the most common complication of extreme prematurity (<28 weeks) (1) and affects approximately 40% of patients (2–5). BPD is characterized by an arrest in lung development that occurs in extremely premature newborns exposed to supplemental oxygen and ventilatory support. In addition to lifelong pulmonary sequalae, BPD is an independent risk factor for abnormal neurodevelopment (6). Even in the absence of the primary reported neonatal brain injuries — intraventricular hemorrhage (IVH) and periventricular leukomalacia (PVL) (7–9) — preterm infants remain at a high risk of lifelong cognitive impairment (10, 11). Specifically, 25% to 50% of extremely preterm infants develop deficits in working memory, learning, and executive function as well as verbal and performance IQ (12–15). Furthermore, children born prematurely are at a higher risk for cerebral palsy (16) and autism spectrum disorders (17). Notably, preterm infants diagnosed with BPD perform significantly more poorly on all neurodevelopmental measures, including cognitive and motor functions, compared with non-BPD preterm born patients (18–21).

Supplemental oxygen administered for respiratory distress is lifesaving, yet it is also one of the main contributors to neonatal morbidity (22, 23) and may play an important role in BPD-associated abnormal neurodevelopment. Preterm infants are particularly vulnerable to elevated oxygen concentrations for three main reasons. First, the fetus’ environment is hypoxic relative to room air (24); therefore, administering oxygen adds to the oxidative stress at birth. Second, preterm infants have not developed the ability to autoregulate their vascular responses to oxygen fluctuations (25–28). Finally, preterm infants have immature antioxidant defenses (24), allowing for high amounts of oxygen and subsequent ROS to reach the developing brain (10).

Neural progenitor cells (NPCs) are crucial for neurodevelopment and cognitive function throughout our lifespan (29–31). Whether NPCs are functionally impaired in preterm infants with BPD remains unexplored. Postnatally, NPCs reside in niches of neurogenesis, i.e., the subventricular zone (SVZ) of the lateral ventricle and the subgranular zone (SGZ) in the dentate gyrus (DG) of the hippocampus (32–37). NPCs are particularly vulnerable to oxidative stress (38–41). Furthermore, NPC fate relies heavily on the surrounding cerebrovasculature (42). The effects of hypoxia on disrupting brain development in cases of apnea (43), hypoxia-ischemic injury (44), IVH (45), and PVL (46) are well established. However, little is known about the effects of hyperoxia on (a) the developing brain vasculature, (b) NPC function, and (c) cognitive performance.

Here, we designed a hyperoxia-induced preclinical model of BPD during early postnatal development in mice and investigated the lifelong implications on the brain vasculature, NPCs, and behavior. We found that experimental postnatal hyperoxia induced a BPD-like lung disease and oxidative stress within the neurogenic niche as well as cerebrovascular deficits. These early perivascular perturbations correlated with NPC functional impairments and, ultimately, permanent cognitive deficits. Furthermore, we identified the dysregulation of key genes involved in cell proliferation and angiogenesis directly following neonatal hyperoxia exposure in neurogenic niches. Long-term follow-up of hyperoxia-exposed mice revealed a transcriptional signature of perturbed vascular autoregulation and neuronal development. These findings establish a relationship between BPD and abnormal neurodevelopment and identify the vascular niche of neurogenesis and NPCs as important targets for future investigations.

## Results

### Hyperoxia-exposed mice maintain arterial oxygen levels, but display lung injury modeling BPD.

**Figure 1:**
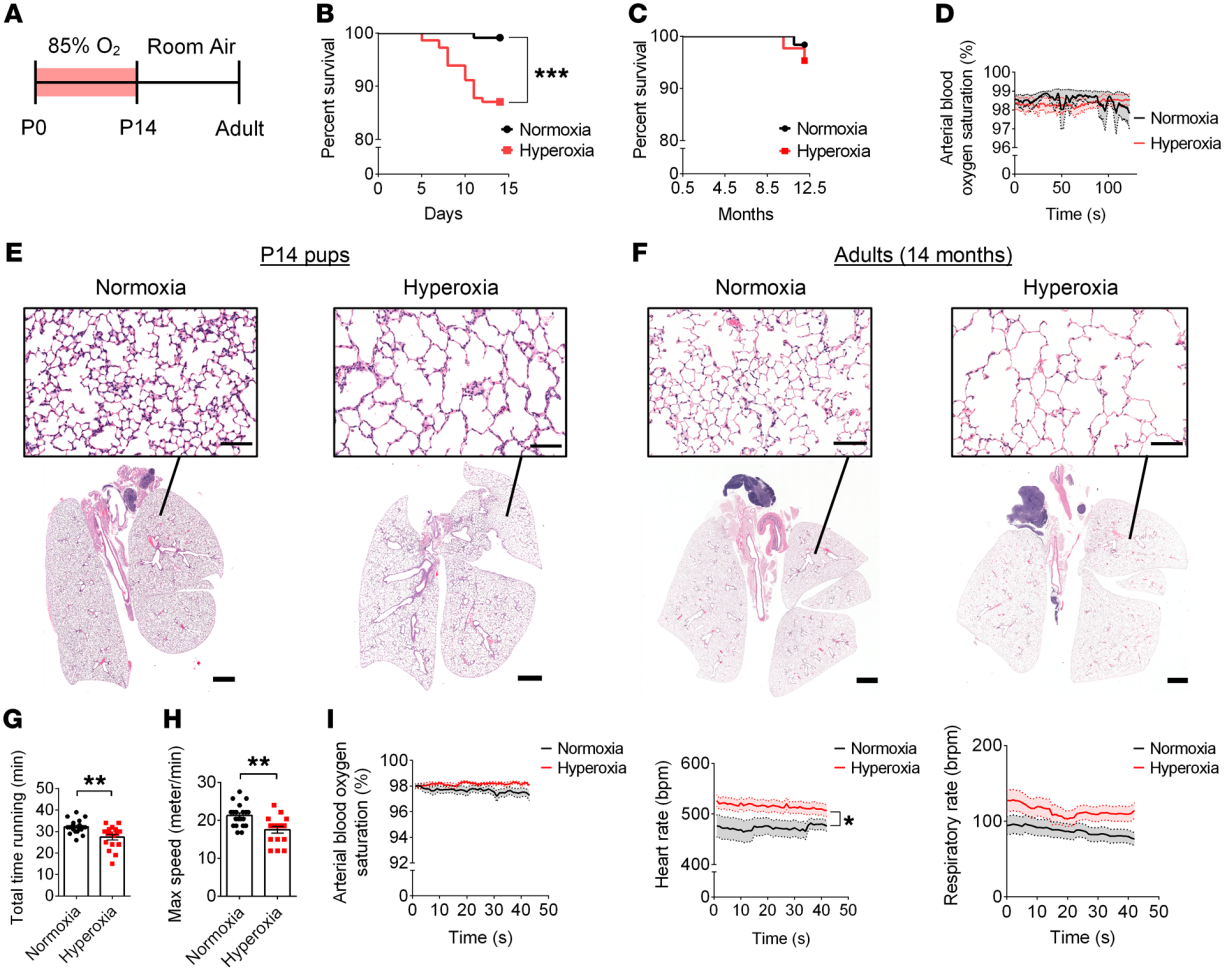
Early developmental hyperoxia exposure induces a lung injury phenotype reminiscent of BPD yet does not lead to arterial hypoxia. (**A**) Representation of the experimental design. WT C57BL/6 mice were exposed to 85% O_2_ from P0 to P14. (**B**) Percentage survival of mice after 14 days of room air exposure (normoxia) or 85% O_2_ exposure (hyperoxia) (normoxia, *n* = 115; hyperoxia, *n* = 147; ***P < 0.001; log-rank test). (**C**) Percentage survival of mice 12 months after exposure to neonatal normoxia or hyperoxia (normoxia, *n* = 62; hyperoxia, *n* = 43; log-rank test). (**D**) Blood oxygen saturation measurements of the femoral artery at rest at P14 (normoxia, *n* = 16; hyperoxia, *n* = 13; 2-way ANOVA with Šidák’s post hoc test for group comparisons). (**E**) Representative images of H&E-stained lung sections of mice at P14. (**F**) Representative H&E-stained lung sections of 14-month-old mice. (**G**) Total duration spent on the metabolic treadmill (normoxia, *n* = 20, hyperoxia, *n* = 17; **P < 0.01; unpaired Student’s *t* test). (**H**) Maximum speed reached on the metabolic treadmill (normoxia, *n* = 20, hyperoxia, *n* = 17; **P < 0.01; unpaired Student’s *t* test). (**I**) Three outcome measurements were assessed for 12- to 14-month-old mice following exercise: blood oxygen saturation of the femoral artery; heart rate (bpm); and respiratory rate (breaths per minute [brpm]) (normoxia, *n* = 20; hyperoxia, *n* = 17; *P < 0.05; 2-way ANOVA with Šidák’s post hoc test for group comparisons). Scale bars: 1,000 μm (full lung sections); 100 μm (high magnification fields of view). Data are represented as mean (solid line) ± SEM (shaded area around line).

To mimic BPD, we exposed C57BL/6 WT mice to 85% O_2_ from P0 to P14 (Figure 1A). At P0, mice are in the saccular stage of lung development and the beginning stages of gliogenesis within the brain, recapitulating extremely preterm human lung (47) and brain development (48). Age-matched control mice were housed in room air. We observed that hyperoxia exposure from P0 to P14 significantly decreased the survival rate of P14 pups, by 13% (Figure 1B). The survival rate from P14 to 12 months of age did not differ significantly (Figure 1C). Therefore, 14 days of neonatal hyperoxia exposure does not affect survival in adulthood.

No difference in the arterial blood oxygen saturation was detected at P14 (Figure 1D), yet hyperoxia-exposed mice showed alveolar rarefication characteristic of BPD (Figure 1E). This was reminiscent of a chronic lung injury, with no signs of recovery as adults (Figure 1F), consistent with recent observations of humans born preterm (49, 50). Next, we tested to determine whether hyperoxia-exposed mice maintained their arterial oxygen levels within a normal physiological range when physically challenged by exercise. Hyperoxia-exposed mice showed impairments on the treadmill compared with normoxia-exposed mice (e.g., running for shorter durations and at a lower maximum speed; Figure 1, G and H). These changes in performance occurred in the absence of differences in arterial oxygen saturation between groups (Figure 1I and Supplemental Figure 1A; supplemental material available online with this article; https://doi.org/10.1172/JCI146095DS1). Metabolic analysis as measured by the comprehensive lab monitoring system (CLAMS) (e.g., oxygen consumption, carbon dioxide production) revealed no differences (Supplemental Figure 1B); however, hyperoxia-exposed mice displayed a significantly increased heart rate and a trend of an increased respiratory rate following exercise (Figure 1I).

### Early postnatal hyperoxia exposure delays brain growth.

The brains of hyperoxia-exposed mice appeared significantly smaller at P14 and 12 months of age (Supplemental Figure 2A). This result was not associated with differences in total body weight at P14 or 12 to 14 months (Supplemental Figure 2A). Hyperoxia-exposed pups had a reduced brain weight even when normalized to body weight (Supplemental Figure 2A). MRI (Supplemental Figure 2B) in adulthood further confirmed the reduction in total brain size in hyperoxia-exposed mice (Supplemental Figure 2, B and C), with an absence of any significant differences in ventricular size (Supplemental Figure 2D). The reduction in total brain size was associated with a significant reduction in hippocampal volume in the hyperoxia-exposed mice (Supplemental Figure 2E). Overall, these results indicate that neonatal hyperoxia exposure leads to a reduction in brain growth during early development that is sustained into adulthood.

### Early postnatal hyperoxia exposure impairs cerebrovascular function.

**Figure 2:**
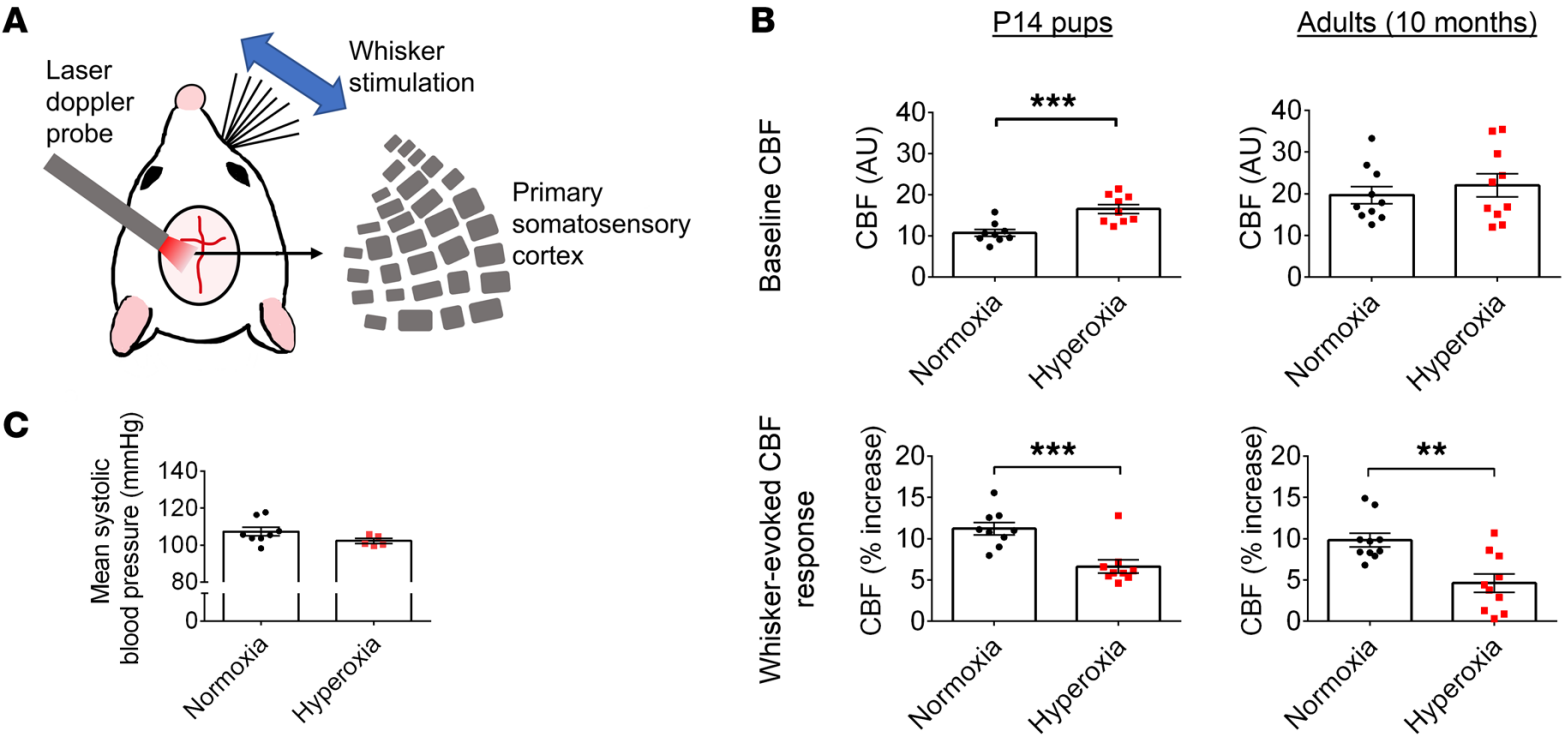
Early developmental hyperoxia exposure leads to neurovascular uncoupling. (**A**) Methods used to measure CBF in P14 and 10-month-old mice. (**B**) Baseline CBF (AU) and percentage change in CBF after whisker stimulation (P14 mice, normoxia, *n* = 9, hyperoxia, *n* = 9; 10-month-old mice, normoxia, *n* = 10, hyperoxia, *n* = 10; **P < 0.01; ***P < 0.001; unpaired Student’s *t* test). (**C**) Mean systolic blood pressure of 10-month-old mice (normoxia, *n* = 8, hyperoxia, *n* = 5; unpaired Student’s *t* test). Data are represented as mean ± SEM.

Increases in neural activity are coupled with increases in cerebral blood flow (CBF) through neurovascular coupling (NVC), a critical process for the blood supply to adapt to the metabolic needs of the surrounding tissue (51–53). The anatomical structure underlying this process, the neurovascular unit (NVU) (51, 54), matures postnatally (52). Here, we tested to determine whether early postnatal hyperoxia affects NVC maturation using minimally invasive laser Doppler flowmetry to measure CBF over the primary somatosensory cortex in anesthetized mice (Figure 2A) (55, 56).

At P14, hyperoxia-exposed mice had higher relative baseline CBF compared with control animals (Figure 2B). There was no baseline difference for adult mice (Figure 2B). To assess NVC, we measured CBF responses to whisker stimulations over the contralateral somatosensory cortex (57). Remarkably, hyperoxia-exposed mice displayed lower evoked CBF responses compared with control mice (Figure 2B). Thus, early hyperoxia resulted in abnormal CBF regulation. However, hyperoxia-exposed adults maintained a systolic blood pressure similar to that of control animals when measured using tail cuffs (Figure 2C), suggesting that the observed CBF impairments do not result from peripheral dysregulation.

### Cerebrovascular alterations in hyperoxia-exposed mice.

**Figure 3:**
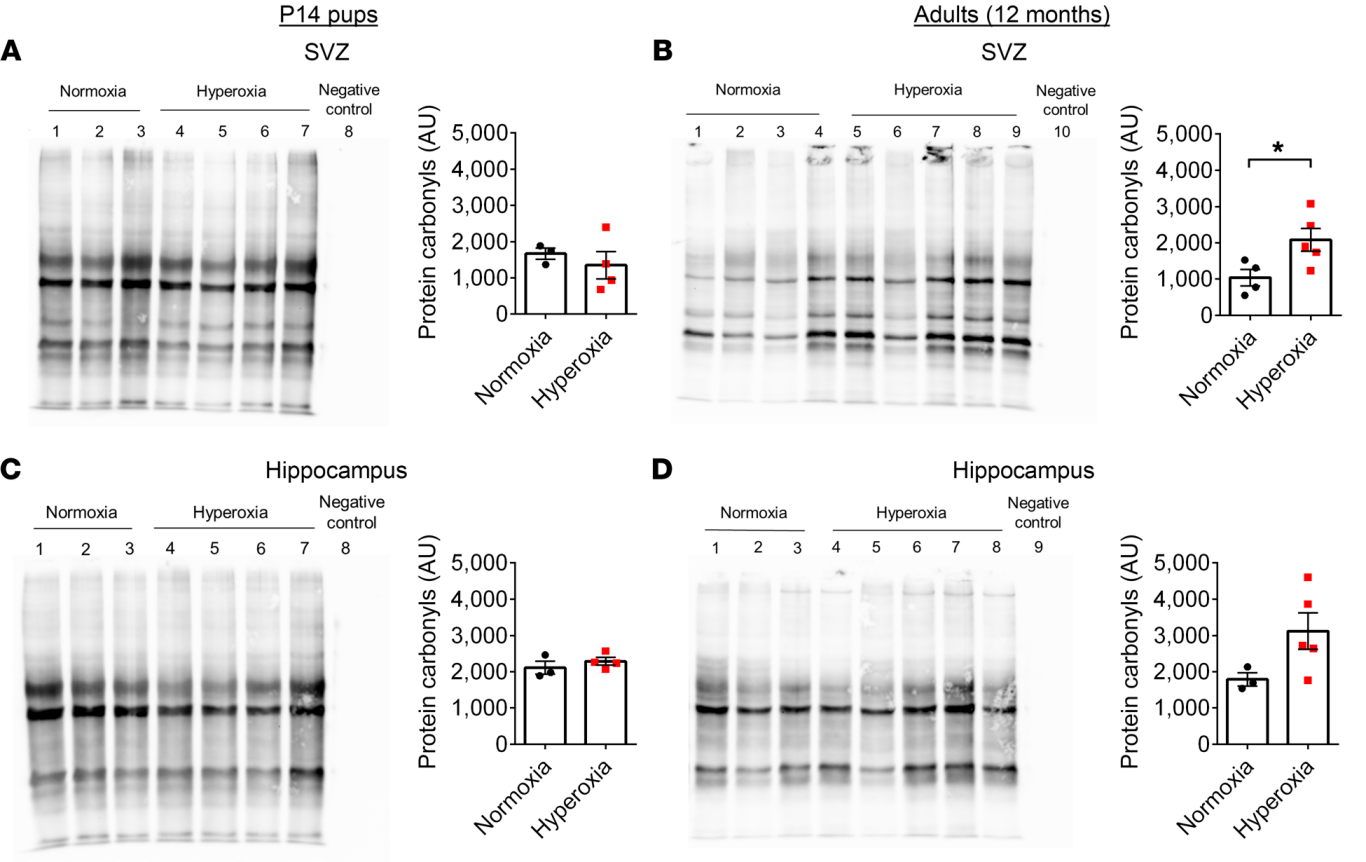
The neurogenic niche regions of hyperoxia-exposed mice show an increase in oxidative stress with age. (**A**) OxyBlot and quantification of protein carbonyls in the SVZ of P14 mice (normoxia, *n* = 3; hyperoxia, *n* = 4; unpaired Student’s *t* test). (**B**) OxyBlot and quantification of protein carbonyls in the SVZ of 12-month-old mice (normoxia, *n* = 4; hyperoxia, *n* = 5; *P < 0.05; unpaired Student’s *t* test). (**C**) OxyBlot and quantification of protein carbonyls in the hippocampus of P14 mice (normoxia, *n* = 3; hyperoxia, *n* = 4; unpaired Student’s *t* test). (**D**) OxyBlot and quantification of protein carbonyls in the hippocampus of 12-month-old mice (normoxia, *n* = 3; hyperoxia, *n* = 5; unpaired Student’s *t* test).

Under normal atmospheric conditions (21% O_2_ at 1 atmosphere), 98% of oxygen is delivered to tissues through binding to hemoglobin in the blood, while 2% is dissolved in the plasma (58). Once the hemoglobin in the blood becomes fully saturated with oxygen, the blood has reached its oxygen-carrying capacity; however, during hyperoxia exposure, the oxygen tension gradient between blood and tissue can significantly increase, leading to tissue hyperoxygenation (59). Considering our observation that early postnatal hyperoxia exposure led to long-term CBF alterations, we tested to determine whether the brain parenchyma displayed oxygen concentration changes. We used an intracerebral oxygen microsensor to measure the oxygen concentration in the primary somatosensory cortex and neurogenic regions in vivo in anesthetized mice. At P14, no differences were found between normoxia- and hyperoxia-exposed mice; however, the brain regions of hyperoxia-exposed mice showed a trend of becoming progressively hyperoxic as the mice aged (Supplemental Figure 3, A–C). To investigate whether this trend led to an increase in oxidative stress in the neurogenic niche regions, we measured protein carbonyls in the SVZ and hippocampus. The addition of carbonyls to proteins occurs in tandem with the level of oxidative reactions (60). OxyBlot analysis revealed no significant differences in protein oxidation in the P14 neurogenic niche regions (Figure 3, A and C). Conversely, at 12 months of age, mice exposed to hyperoxia during the neonatal period demonstrated significantly increased protein oxidation in the SVZ and a trend of increased protein oxidation in the hippocampus (Figure 3, B and D). These findings provide strong quantitative evidence that the neonatal hyperoxia exposure in mice leads to a substantial increase in oxidative stress in the neurogenic niche during adulthood. For a full list of antibodies, see Supplemental Table 1.

**Figure 4:**
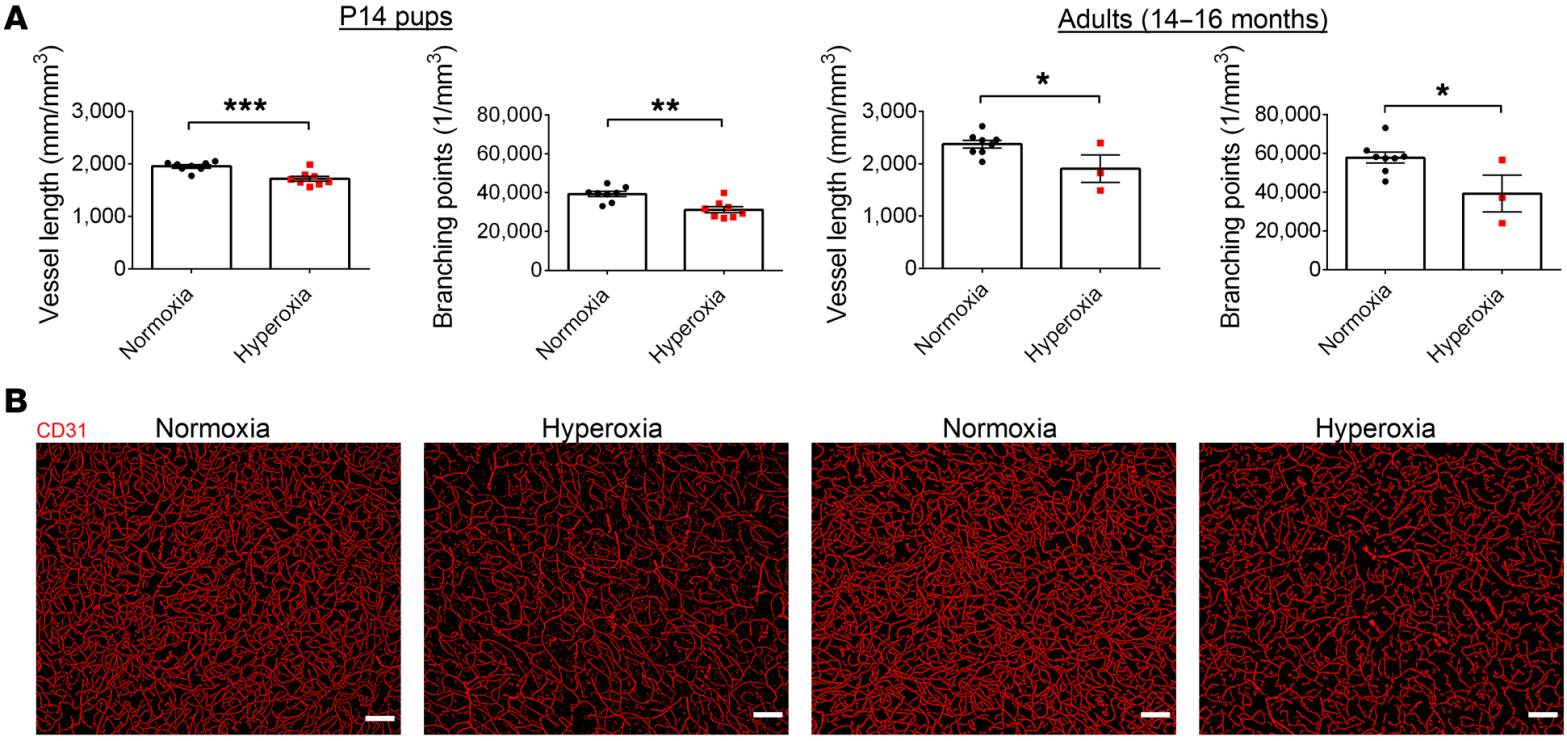
Neurovascular uncoupling within the cortex of hyperoxia-exposed mice correlates with significant vascular remodelling. (**A**) Average vessel length and number of branching points from cortical layers II/III, IV, and V for P14 and 14- to 16-month-old mice (P14 mice, normoxia, *n* = 8, hyperoxia, *n* = 8; 14- to 16-month-old mice, normoxia, *n* = 8, hyperoxia, *n* = 3; *P < 0.05; **P < 0.01; ***P < 0.001, unpaired Student’s *t* test). (**B**) Representative images of cortical layers in P14 and 14- to 16-month-old mice. Scale bars: 100 μm. Data are represented as mean ± SEM.

We next asked whether the relatively hyperoxic microenvironment was associated with vascular structural changes within the brain during this critical period of development. To test this, we used an approach combining immunofluorescent staining of endothelial networks and automatic computational analysis of 3D images/reconstructions for unbiased quantification of cerebrovascular density and branching in the cortex. We identified vascular remodeling in the cortex of hyperoxia-exposed mice. Neonatal hyperoxia exposure led to reduced vessel length and branch point number early in life that continued into adulthood (Figure 4, A and B, and Supplemental Figure 4). Thus, hyperoxia exposure during early postnatal development in mice leads to long-term vascular structural impairments in the cerebral cortex.

### Hyperoxia-induced reduction in NPCs is associated with vascular remodeling.

**Figure 5:**
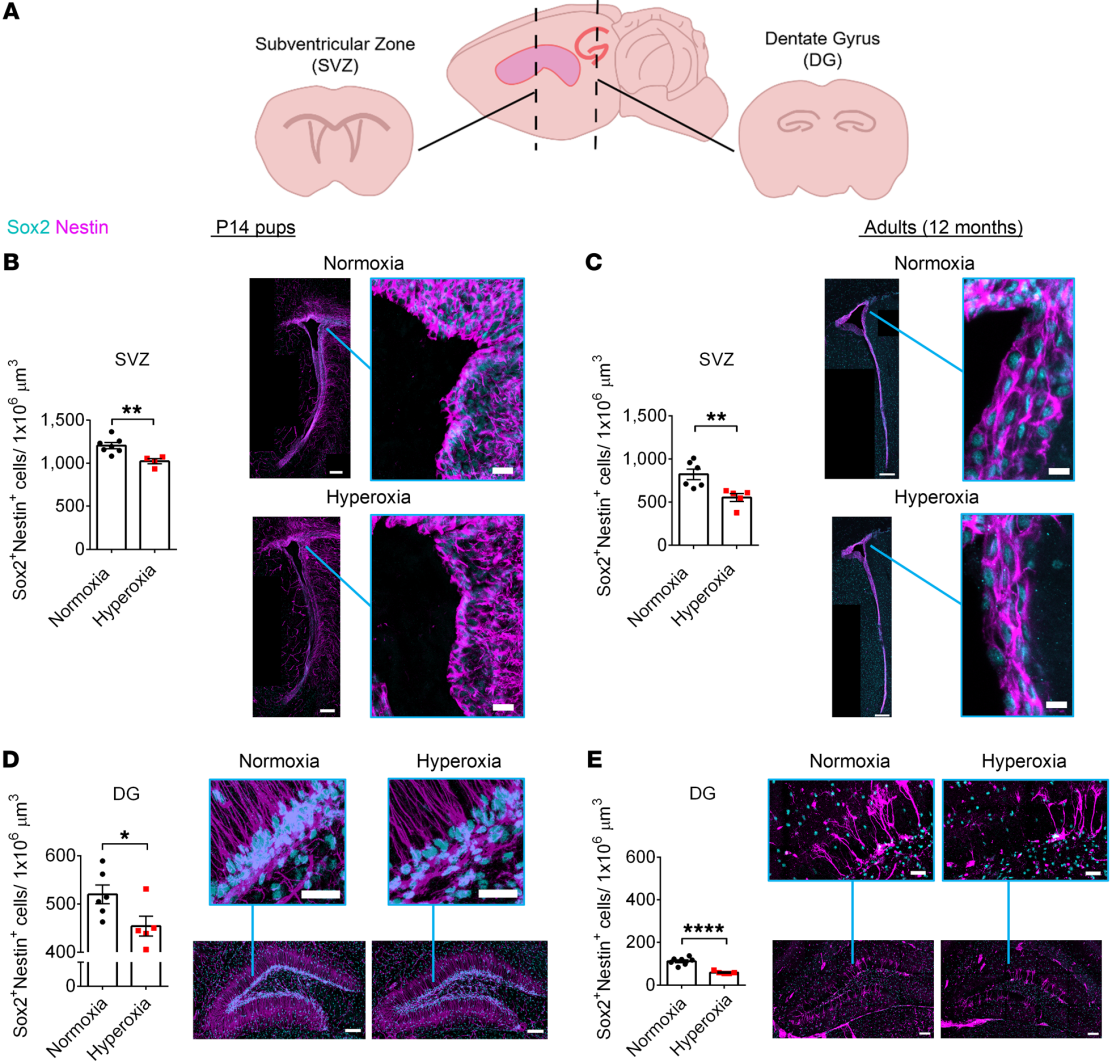
Early developmental hyperoxia exposure leads to long-term NPC reduction. (**A**) Schematic of the NPC niche regions, the SVZ and DG. (**B** and **C**) Quantification and representative images of NPCs (Sox2^+^, nestin^+^) in the SVZ of P14 mice (**B**, normoxia, *n* = 7, hyperoxia, *n* = 4; unpaired Student’s *t* test) and 12-month-old mice (**C**, normoxia, *n* = 6, hyperoxia, *n* = 5; unpaired Student’s *t* test). **P < 0.01. Scale bars: 150 μm (whole ventricle); 20 μm (magnified images, P14 mice); 12 μm (magnified images, 12-month-old mice). (**D** and **E**) Quantification and representative images of NPCs (Sox2^+^, nestin^+^) in the DG of P14 mice (**D**, normoxia, *n* = 6, hyperoxia, *n* = 5; *P < 0.05; unpaired Student’s *t* test) and 12-month-old mice (**E**, normoxia, *n* = 7, hyperoxia, *n* = 5; ****P < 0.0001; unpaired Student’s *t* test). Scale bars: 100 μm (whole DG); 30 μm (magnified images). Data are represented as mean ± SEM.

To determine the effect of early life hyperoxia on NPCs, we quantified NPCs (Sox2^+^, nestin^+^ cells) in the neurogenic niche regions, the SVZ and DG (Figure 5A), of P14 and 12-month-old mice. Animals exposed to hyperoxia showed significantly fewer NPCs in the SVZ and DG compared with control mice at P14 and 12 months (Figure 5, B–E). At P14, hyperoxia-exposed mice had an average of approximately 15% fewer NPCs in the SVZ and DG than control mice. At 12 months, hyperoxia-exposed mice had an average of 33% fewer NPCs located within the SVZ and 47% fewer NPCs located within the DG compared with normoxia-exposed mice. Therefore, neonatal hyperoxia exposure leads to a significant long-term NPC population decline (*P* < 0.01).

**Figure 6:**
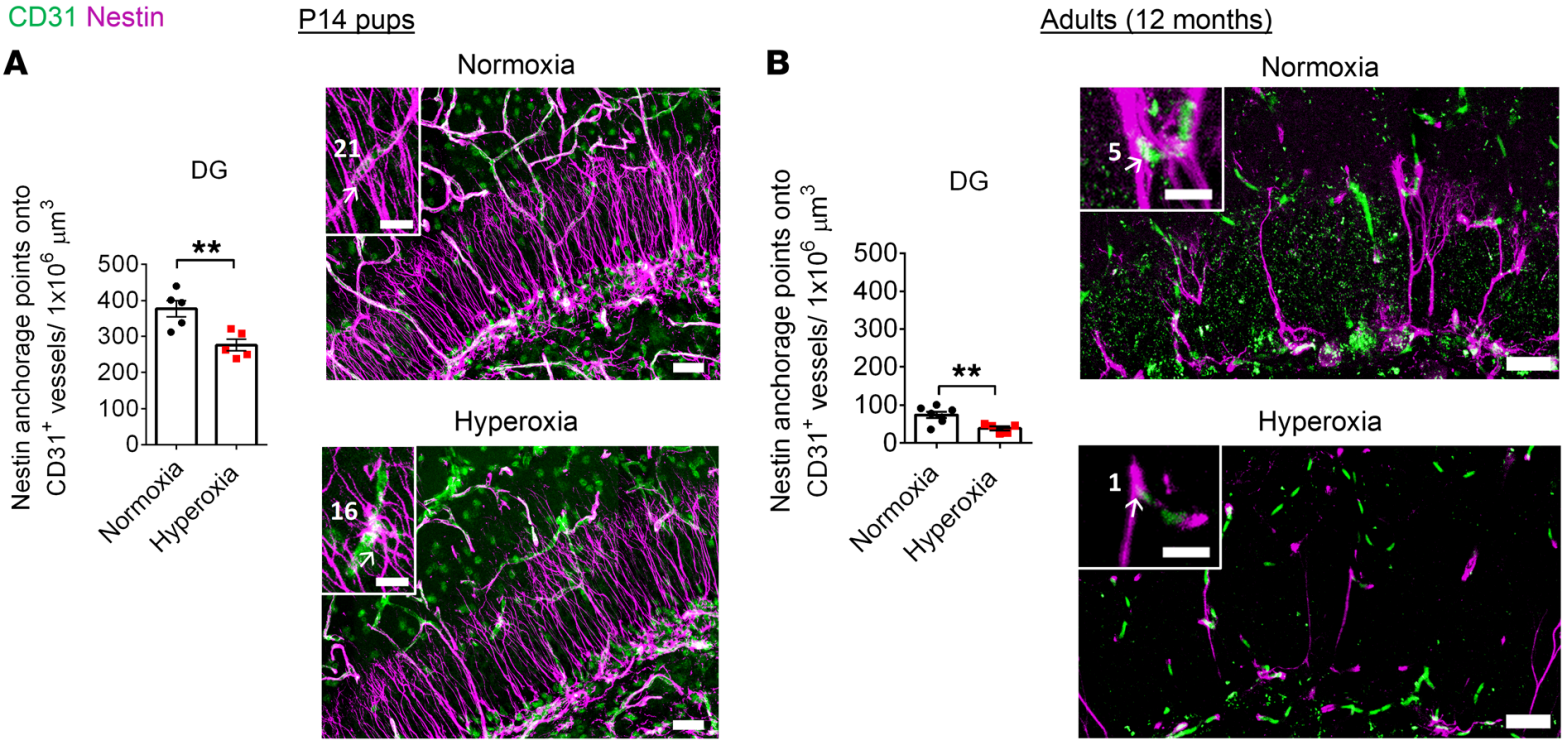
Long-term reduction in NPC anchorage to CD31^+^ vessels in the DG of hyperoxia-exposed mice. (**A** and **B**) Quantification and representative images of the anchorage points of NPC processes (nestin^+^) connecting to vascular ECs (CD31^+^) in the DG of P14 mice (**A**, normoxia, *n* = 5; hyperoxia, *n* = 5; unpaired Student’s *t* test, **P < 0.01) and 12-month-old mice (**B**, normoxia, *n* = 7; hyperoxia, *n* = 5; unpaired Student’s *t* test, **P < 0.01). Scale bars: 30 μm (lower magnification images); 12 μm (magnified images, P14 DG); 10 μm (magnified images, 12-month DG). White arrows indicate a nestin anchorage point onto CD31^+^ vessels. White numbers indicate total number of anchorage points in the representative field of view. Data are represented as mean ± SEM.

We next assessed the vasculature within the DG. The physical and molecular crosstalk between vascular endothelial cells (ECs) and NPCs within neurogenic niches guides NPC maturation and function (42). To examine this relationship in the context of hyperoxia, we investigated the anchorage of NPC processes (nestin^+^) onto ECs (CD31^+^). On average, P14 hyperoxia-exposed mice had 27% fewer anchorage points compared with control mice (Figure 6A). This dramatic phenotype became more pronounced with age, as 12-month-old hyperoxia-exposed mice showed an average of 48% fewer anchorage points compared with normoxia-exposed control mice (Figure 6B). Thus, after neonatal hyperoxia exposure, NPCs lack the essential scaffold provided by the vascular niche necessary to maintain the postnatal NPC population.

### Early postnatal hyperoxia exposure perturbs key pathways involved in proliferation, angiogenesis, neurotransmission, and vascular autoregulation.

**Figure 7:**
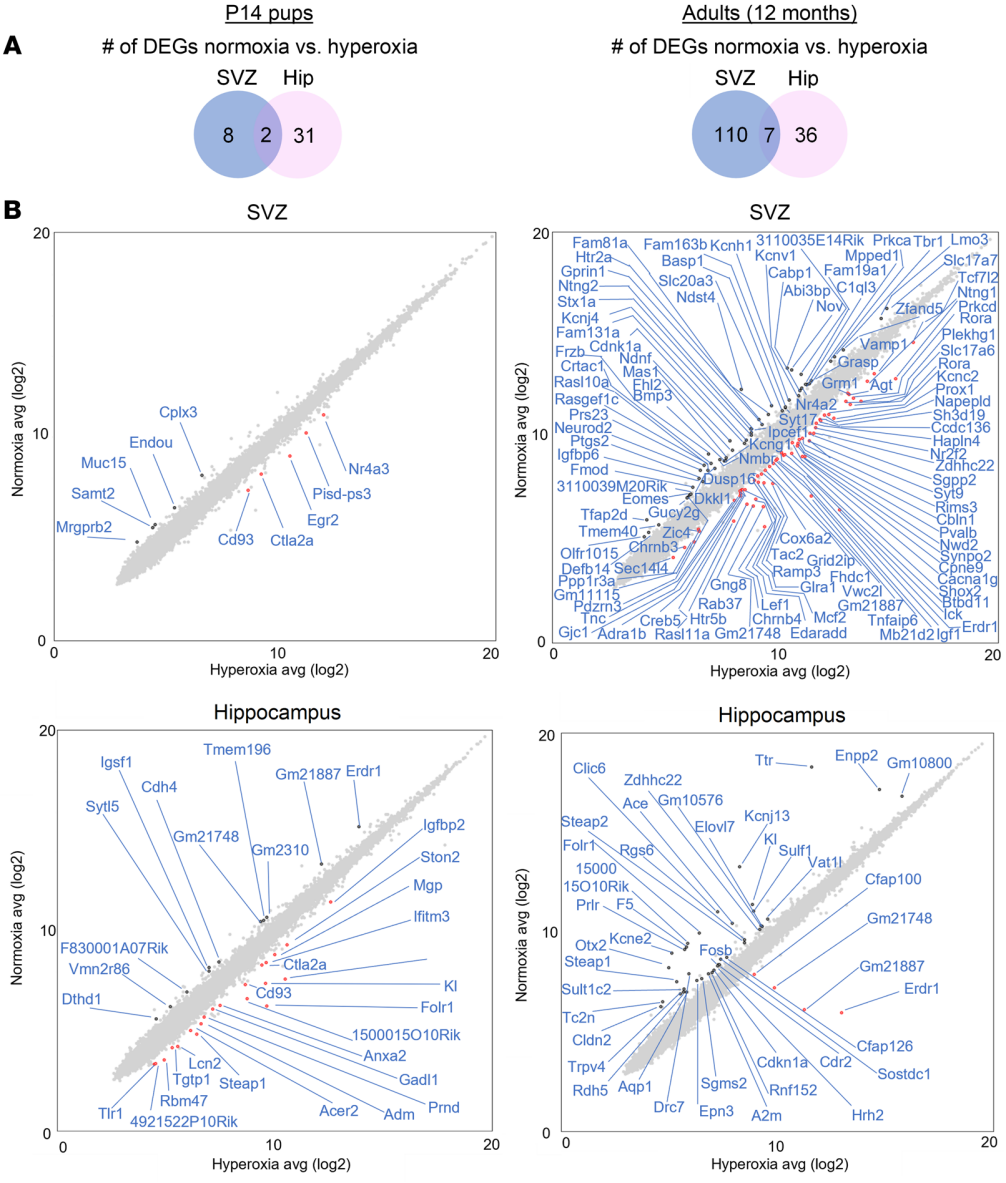
Neonatal hyperoxia exposure leads to varying transcriptional signatures dependent on brain region and age. (**A**) Quantity of DEGs filtered by a fold change of more than 2 or less than –2 and a *P* value of < 0.05. (**B**) DEGs for the SVZ and hippocampus of P14 pups and 12-month-old adults exposed to normoxia versus hyperoxia in early life.

To gain a better understanding of the molecular mechanisms involved in the hyperoxia-induced vascular and NPC deficits, we conducted transcriptome-wide gene expression profiling of the neurogenic niche regions in P14 and 12-month-old mice. Parameters to identify differentially expressed genes (DEG) included a fold change of more than 2 or less than –2 and a *P* value of less than 0.05. Analysis of over 20,000 genes identified 10 DEGs in the SVZ of P14 mice, 33 DEGs in the hippocampus of P14 mice, 117 DEGs in the SVZ of 12-month-old mice, and 43 DEGs in the hippocampus of 12-month-old mice (Figure 7A). Importantly, cytotoxic T lymphocyte–associated protein 2 α (*Ctla2a*), a strong inhibitor of angiogenesis (61), was significantly upregulated in the SVZ (*P* < 0.01) and hippocampus (*P* < 0.0001) of hyperoxia-exposed P14 pups. Furthermore, in the P14 SVZ of hyperoxia-exposed mice, there was an upregulation of nuclear receptor subfamily 4 group A member 3 (*Nr4a3*), a transcriptional target of p53 that inhibits proliferation (62). In the P14 hippocampus of hyperoxia-exposed mice, there was a significant upregulation of RNA-binding motif protein 47 (*Rbm47*), another gene known to inhibit cell proliferation (Figure 7B). Interestingly, over time, the transcriptional signatures of the neurogenic niche regions changed. In the 12-month-old SVZ and hippocampus of hyperoxia-exposed mice, there was a significant increase in the expression of erythroid differentiation regulator 1 (*Erdr1*). *Erdr1* has not been well characterized in brain tissue, but has been shown to inhibit proliferation of cancerous cells (63). The SVZ of 12-month-old normoxia- versus hyperoxia-exposed mice showed the greatest transcriptional differences compared with those at the P14 time point and in the hippocampal brain region. Of note, key genes identifying NPCs (eomesodermin [*Eomes*]/T-box brain protein 2 [*Tbr2*] and postmitotic neurons [*Tbr1*]) were significantly downregulated following postnatal hyperoxia exposure (*P* < 0.05).

**Figure 8:**
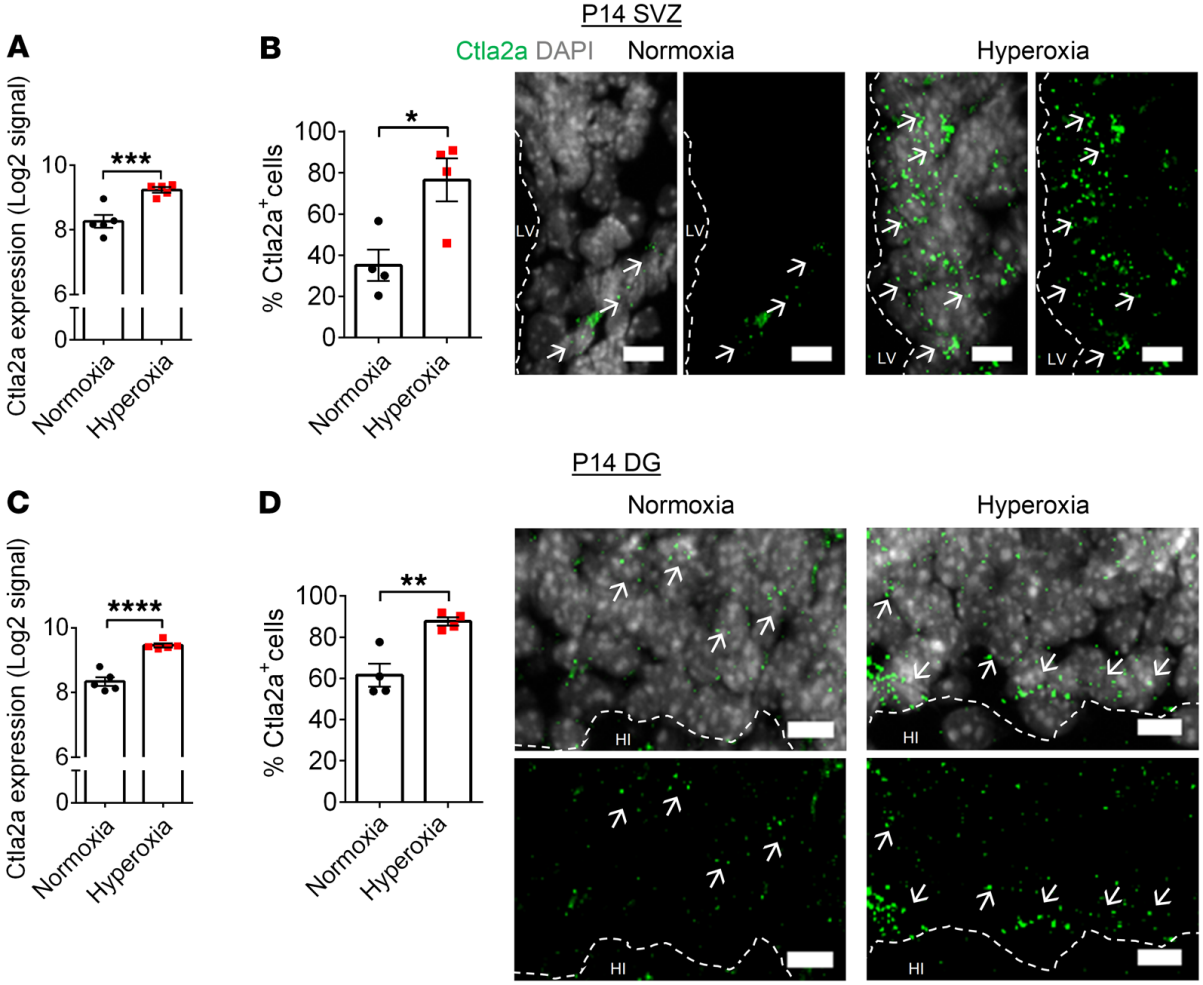
Early developmental hyperoxia exposure leads to increased *Ctla2a* expression in neurogenic niche regions. (**A**) Microarray analysis of *Ctla2a* expression in the SVZ of P14 mice (normoxia, *n* = 5; hyperoxia, *n* = 5; ***P < 0.001; empirical Bayes test). (**B**) Quantification and representative images of *Ctla2a* expression in the SVZ of P14 mice (normoxia, *n* = 4, hyperoxia, *n* = 4; *P < 0.05; unpaired Student’s *t* test). (**C**) Microarray analysis of *Ctla2a* expression in the DG of P14 mice (normoxia, *n* = 5; hyperoxia, *n* = 5; ****P < 0.0001; empirical Bayes test). (**D**) Quantification and representative images of *Ctla2a* expression in the DG of P14 mice (normoxia, *n* = 4, hyperoxia, *n* = 4; **P < 0.01; unpaired Student’s *t* test). Scale bars: 10 μm. Representative images show the combined *Ctla2a* and DAPI channels as well as the individual *Ctla2a* channel. White arrows indicate examples of *Ctla2a^+^* cells. Data are represented as mean ± SEM. LV, lateral ventricle; HI, hilus.

We conducted FISH to validate the upregulation of *Ctla2a* expression, a potential key player in the inhibition of angiogenesis during early development of hyperoxia-exposed mice. Analysis confirmed that there was a significant increase in *Ctla2a*^+^ cells within neurogenic niche regions of hyperoxia-exposed pups compared with normoxia-exposed control animals (Figure 8).

**Figure 9:**
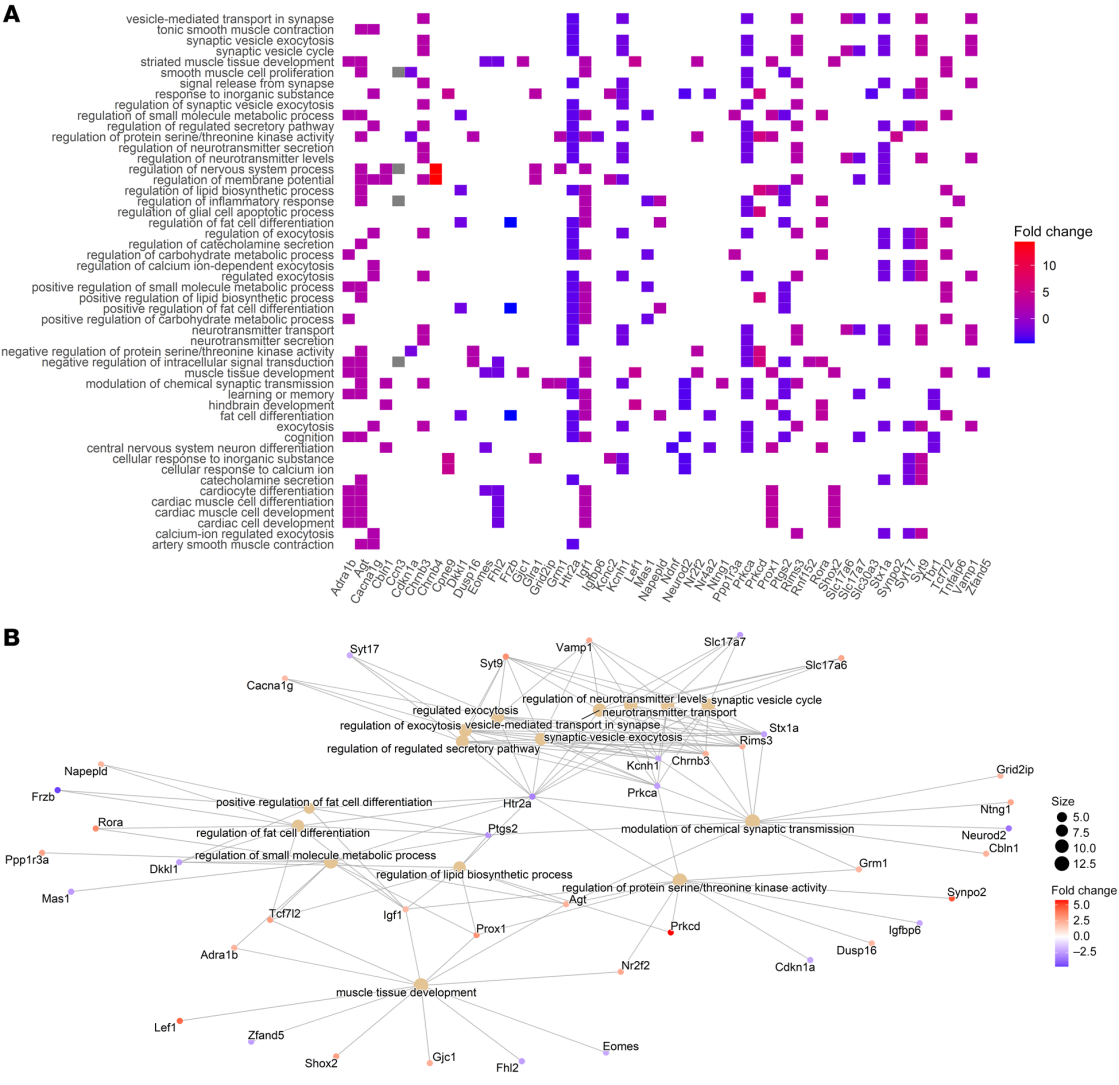
GO enrichment analysis of SVZ tissue from 12-month-old mice reveals that early life hyperoxia exposure leads to long-term transcriptional changes involved in many biological processes, including vascular autoregulation, brain development, and neurotransmission. (**A**) Heatmap plot of the top 50 enrichment terms from the SVZ tissue of normoxia- versus hyperoxia-exposed 12-month-old mice. (**B**) Network plot of the of the top 15 enrichment terms from the SVZ tissue of normoxia- versus hyperoxia-exposed 12-month-old mice.

**Figure 10:**
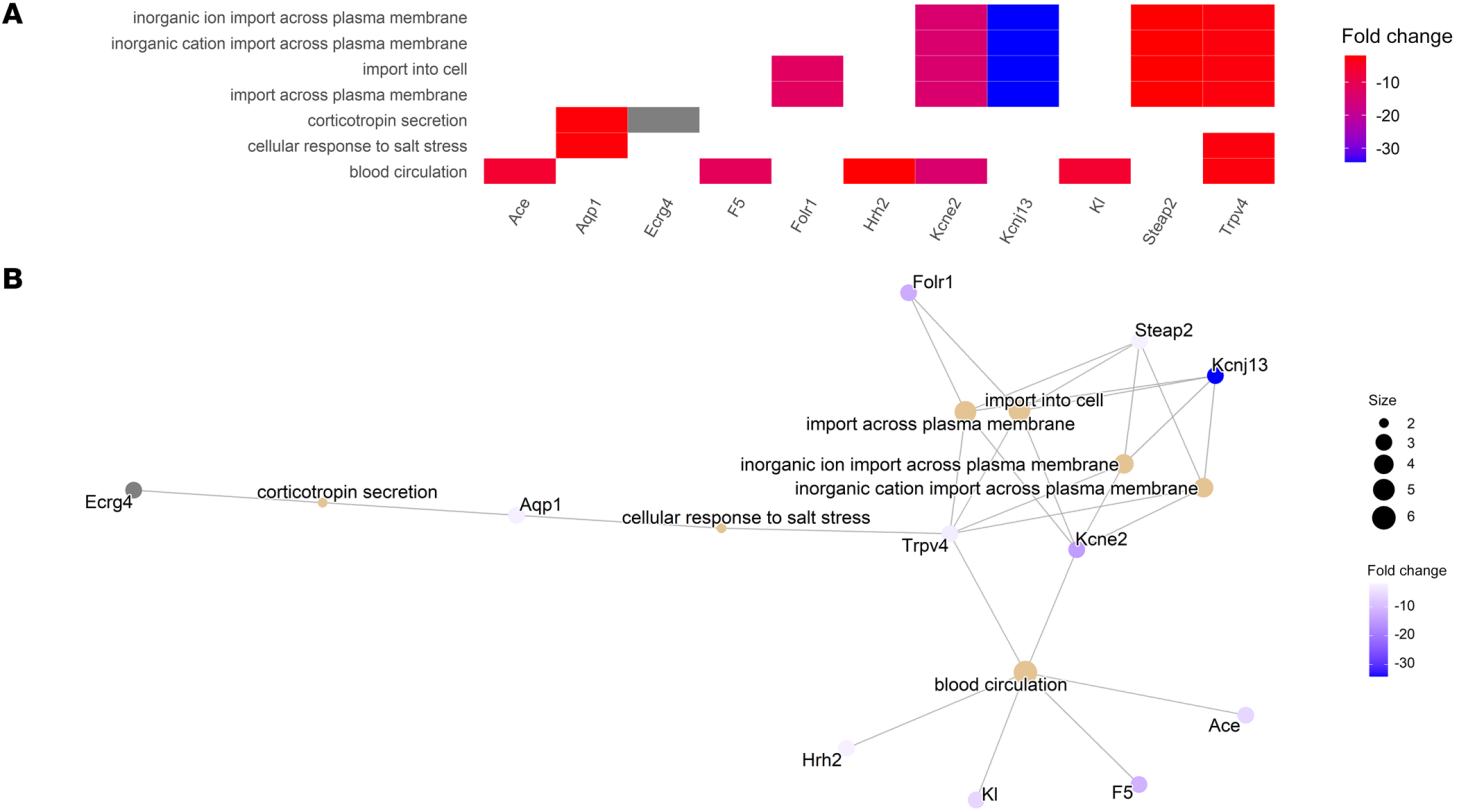
GO enrichment analysis of hippocampal tissue from 12-month-old mice reveals that early life hyperoxia exposure leads to long-term transcriptional changes involved in biological processes relating to vascular autoregulation, transfer across plasma membranes, and response to salt stress. (**A**) Heatmap plot of the enrichment terms from the hippocampal tissue of normoxia- versus hyperoxia-exposed 12-month-old mice. (**B**) Network plot of the enrichment terms from the hippocampal tissue of normoxia- versus hyperoxia-exposed 12-month-old mice.

Gene ontology (GO) analysis revealed many dysregulated pathways in the SVZ of hyperoxia-exposed 12-month-old mice, including regulation of neurotransmitter signaling, neuronal differentiation, and artery smooth muscle contraction (Figure 9, A and B). GO analysis of 12-month-old hippocampal gene expression demonstrated that early life hyperoxia exposure led to long-term perturbation in pathways involved in blood circulation, transport across the plasma membrane, and response to salt stress (Figure 10, A and B). For a full list of characterized DEGs with associated neural functions, see Supplemental Tables 2–5.

### Long-term NPC functional impairment following postnatal hyperoxia exposure.

**Figure 11:**
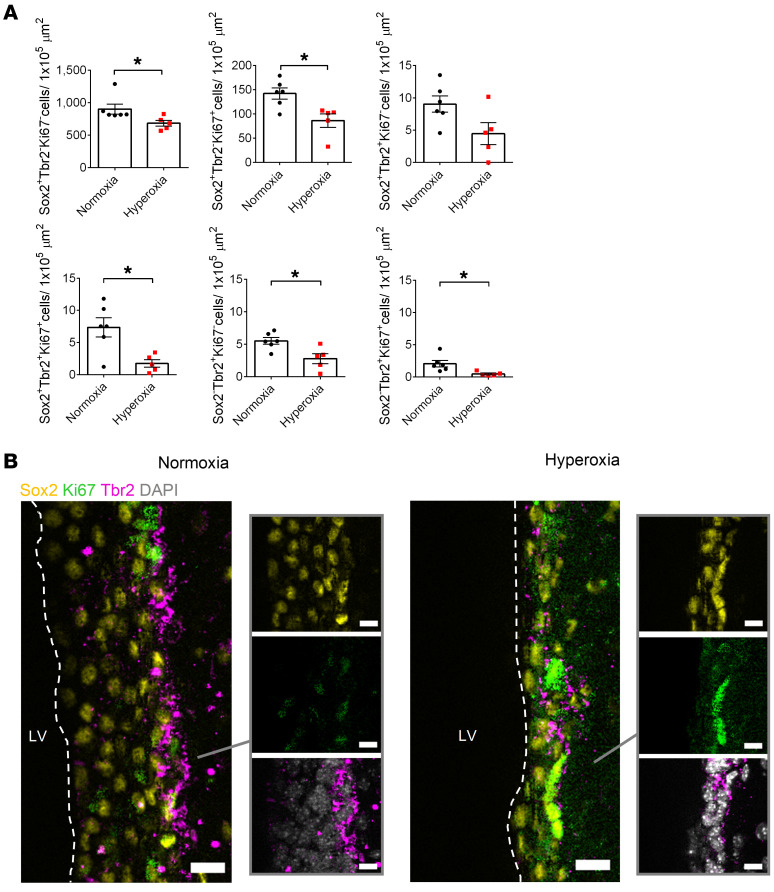
Neonatal hyperoxia exposure leads to a long-term reduction in the SVZ neural stem and progenitor population and impairs these cells’ ability to proliferate during adulthood. (**A**) Quantification of type B neural stem cells (Sox2^+^Tbr2^–^Ki67^–^), proliferating type B neural stem cells (Sox2^+^Tbr2^–^Ki67^+^), immature type C NPCs (Sox2^+^Tbr2^+^Ki67^–^), proliferating immature type C NPCs (Sox2^+^Tbr2^+^Ki67^+^), mature type C NPCs (Sox2^–^Tbr2^+^Ki67^–^), and proliferating mature type C NPCs (Sox2^–^Tbr2^+^Ki67^+^) in the SVZ of 12-month-old mice (normoxia, *n* = 6; hyperoxia *n* = 5; *P < 0.05; unpaired Student’s *t* test). (**B**) Representative images of the SVZ region of normoxia- versus hyperoxia-exposed mice. Scale bars: 15 μm (composite images); 10 μm (single-channel images). Data are represented as mean ± SEM.

We sought to further characterize the NPC population in the SVZ following postnatal hyperoxia by quantifying type B neural stem cells (Sox2^+^Tbr2^–^), immature type C NPCs (Sox2^+^Tbr2^+^), and mature type C NPCs (Sox2^–^Tbr2^+^) that were proliferative (Ki67^+^) or nonproliferative (Ki67^–^). At P14, we found that hyperoxia-exposed mice had a significant reduction in proliferative neural stem cells (Supplemental Figure 5). At 12 months of age, we found a trend of reduced immature type C NPCs and a significant reduction in all other NPC populations (both proliferative and nonproliferative) in hyperoxia-exposed mice (Figure 11, A and B). This provides confirmation of the gene expression data showing that there is long-term *Tbr2* downregulation following postnatal hyperoxia exposure and evidence of an impairment in the ability of NPCs of hyperoxia-exposed mice to proliferate during adulthood.

**Figure 12:**
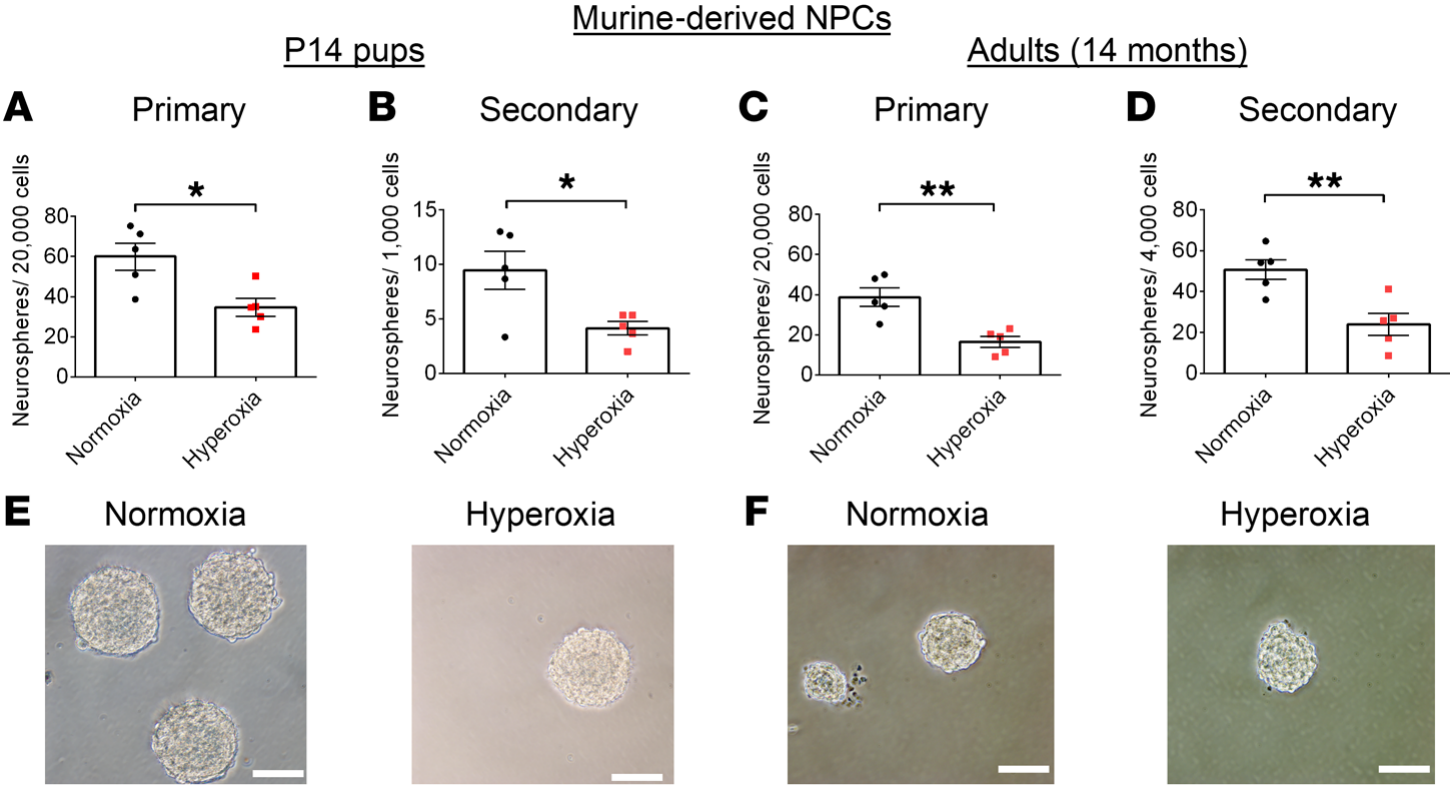
Hyperoxia-induced NPC reduction is associated with impaired NPC self-renewal. (**A**–**D**) Quantification of neurospheres formed by NPCs from the SVZ of P14 (**A** and **B**) and 14-month-old (**C** and **D**) mice (normoxia, *n* = 5; hyperoxia, *n* = 5; *P < 0.05, **P < 0.01; unpaired Student’s *t* test). (**E** and **F**) Representative images of neurospheres formed by NPCs from P14 (**E**) and 14-month-old (**F**) mice. Scale bars: 100 μm. Data are represented as mean ± SEM.

To determine whether an impairment in intrinsic NPC self-renewal capabilities may be contributing to the reduced NPC population in hyperoxia-exposed mice, we isolated NPCs from the SVZ of P14 and 14-month-old mice and conducted neurosphere assays. Hyperoxia exposure led to fewer neurospheres at P14 (Figure 12, A and B) and, remarkably, also at 14 months (Figure 12, C and D). There were no differences between groups in terms of neurosphere size (Figure 12, E and F, and Supplemental Figure 6, A–H). This indicates that the ability of NPCs to form new neurospheres, i.e., self-renewal, is impaired in hyperoxia-exposed mice.

**Figure 13:**
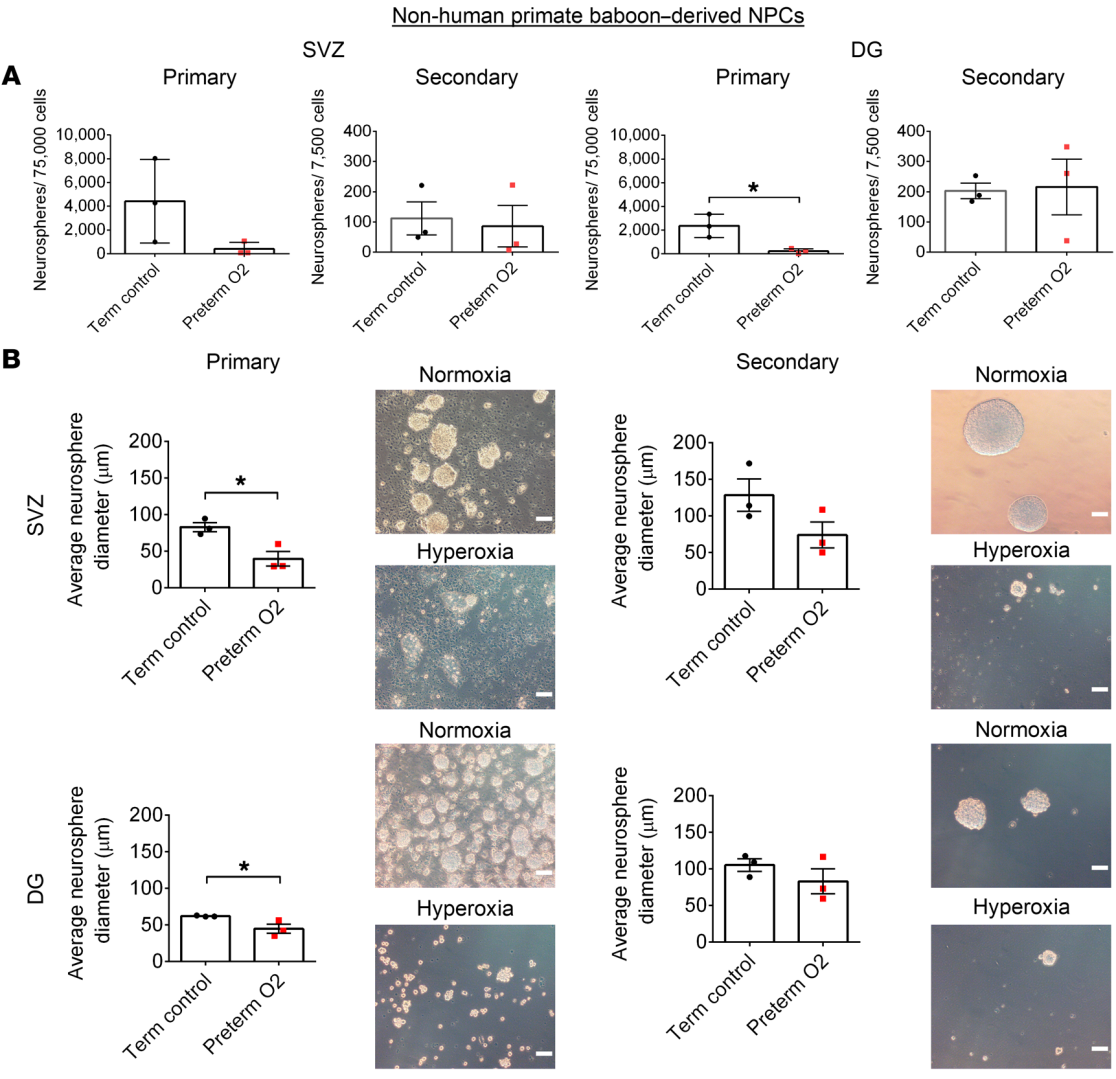
Hyperoxia-exposed preterm baboon–derived NPCs form fewer and smaller neurospheres compared with term control NPCs. (**A**) Quantification of primary and secondary neurospheres formed by NPCs from the SVZ and DG of neonatal baboons (term control, *n* = 3; preterm O_2_, *n* = 3; *P < 0.05; unpaired Student’s *t* test). (**B**) Quantification and representative images of the average primary and secondary neurosphere diameter formed by NPCs from the SVZ and DG of neonatal baboons (term control, *n* = 3; preterm O_2_, *n* = 3; *P < 0.05; unpaired Student’s *t* test). Scale bars: 100 μm. Data are represented as mean ± SEM.

To confirm our findings, we conducted similar studies with nonhuman primates (baboons). This model more closely mimics the preterm human condition, as baboons share many biological and genetic characteristics with humans (64). To this end, we isolated NPCs from the SVZ and DG of extremely preterm and term neonatal baboons, an animal model that has previously been shown to recapitulate human preterm birth complications (65–67). NPCs isolated from preterm neonatal baboons and exposed to 60% O_2_ in vitro for 48 hours formed fewer neurospheres (Figure 13A) and smaller neurospheres (Figure 13B) compared with NPCs from control (term) baboons, although these effects appeared transient. The baboon cells showed greater variability between samples compared with murine cells, likely due to the greater genetic variability in the baboon population. It is also clear that a combination of hyperoxia and vascular deficits in vivo contributes to a more robust impaired NPC phenotype than hyperoxia alone in vitro. Overall, these findings demonstrate that hyperoxia exposure in early developmental life in 2 independent preclinical models leads to deficits in the NPC population.

**Figure 14:**
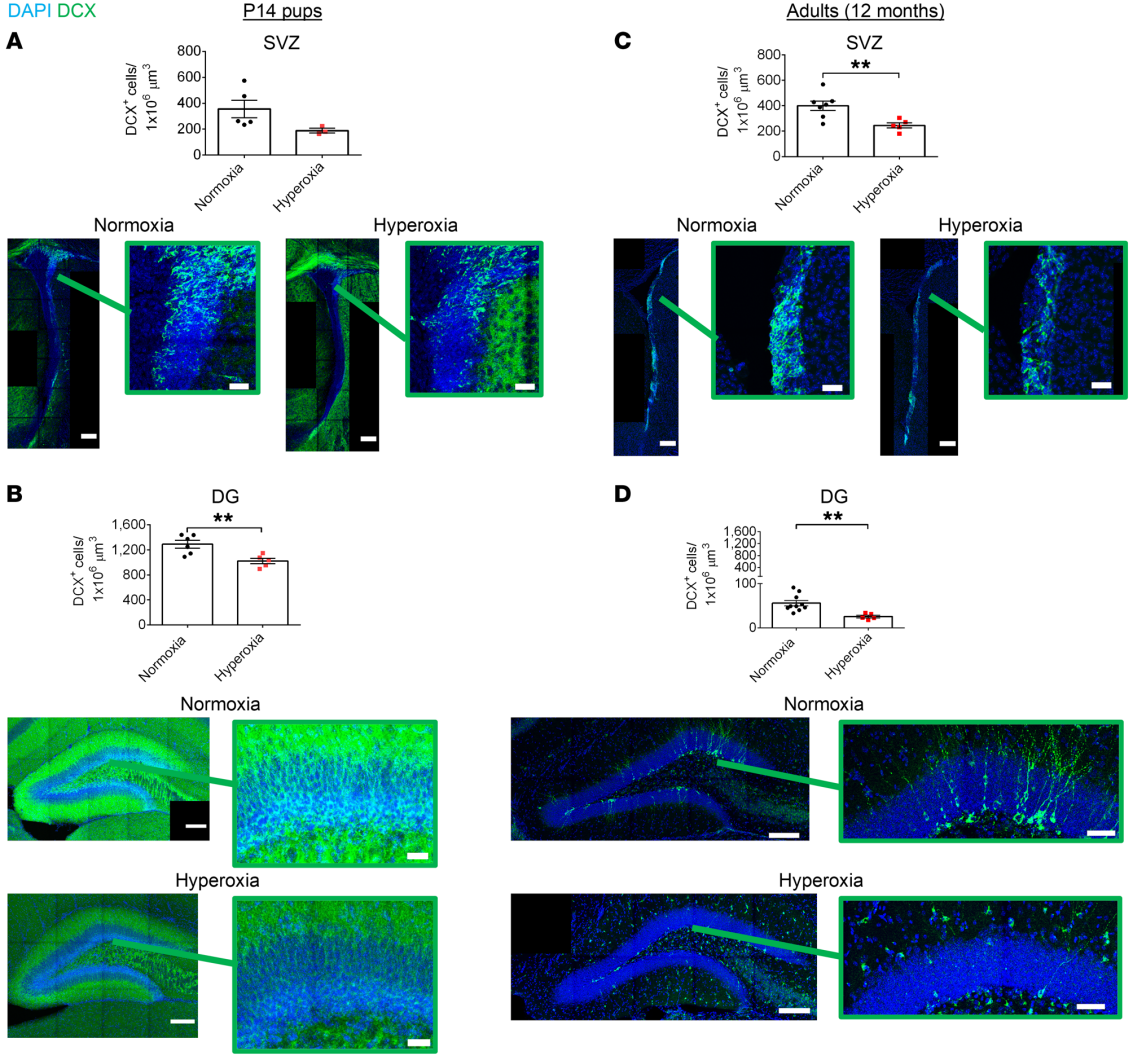
Early developmental hyperoxia exposure leads to reduced neurogenesis that persists into adulthood. (**A**) Quantification and representative images of newborn neurons (DCX^+^) in the SVZ of P14 mice (normoxia, *n* = 5; hyperoxia, *n* = 3; Student’s *t* test). Scale bars: 150 μm (whole ventricles); 30 μm (magnified images). (**B**) Quantification and representative images of newborn neurons (DCX^+^) in the DG of P14 mice (normoxia, *n* = 6; hyperoxia, *n* = 5; **P < 0.01; unpaired Student’s *t* test). Scale bars: 150 μm (whole DG); 30 μm (magnified images). (**C**) Quantification and representative images of newborn neurons (DCX^+^) in the SVZ of 12-month-old mice (normoxia, *n* = 7; hyperoxia, *n* = 5; **P < 0.01; unpaired Student’s *t* test). Scale bars: 150 μm (whole ventricles); 30 μm (magnified images). (**D**) Quantification and representative images of newborn neurons (DCX^+^) in the DG of 12-month-old mice (normoxia, *n* = 10; hyperoxia, *n* = 5; **P < 0.01; unpaired Student’s *t* test). Scale bars: 150 μm (whole DG); 50 μm (magnified images). Data are represented as mean ± SEM.

We hypothesized that the decline in the NPC population would result in an overall reduction in the generation of adult-born neurons. The NPCs of the SVZ differentiate to immature neurons (neuroblasts) that migrate along the rostral migratory stream to produce neurons in the olfactory bulb (68). NPCs of the DG differentiate into neuroblasts and form new granule neurons within the DG (36). We identified newborn neurons with doublecortin (DCX). At P14, we found that hyperoxia-exposed mice had reduced DCX^+^ cells in the SVZ and significantly reduced DCX^+^ cells in the DG (*P* < 0.01) (Figure 14, A and B). Of note, investigation of 12-month-old mice demonstrated that hyperoxia-exposed animals had significantly impaired neurogenesis in adulthood in both regions (*P* < 0.01) (Figure 14, C and D). Therefore, these data demonstrate reduced neurogenesis in NPC niches from hyperoxia-exposed mice. This is in alignment with the reduced hippocampal volume measured via MRI (Supplemental Figure 2E), and thus, limited neurogenesis after early life hyperoxia exposure may contribute to a smaller hippocampal structure.

### Hyperoxia exposure during early postnatal development is associated with long-term motor and cognitive deficits.

**Figure 15:**
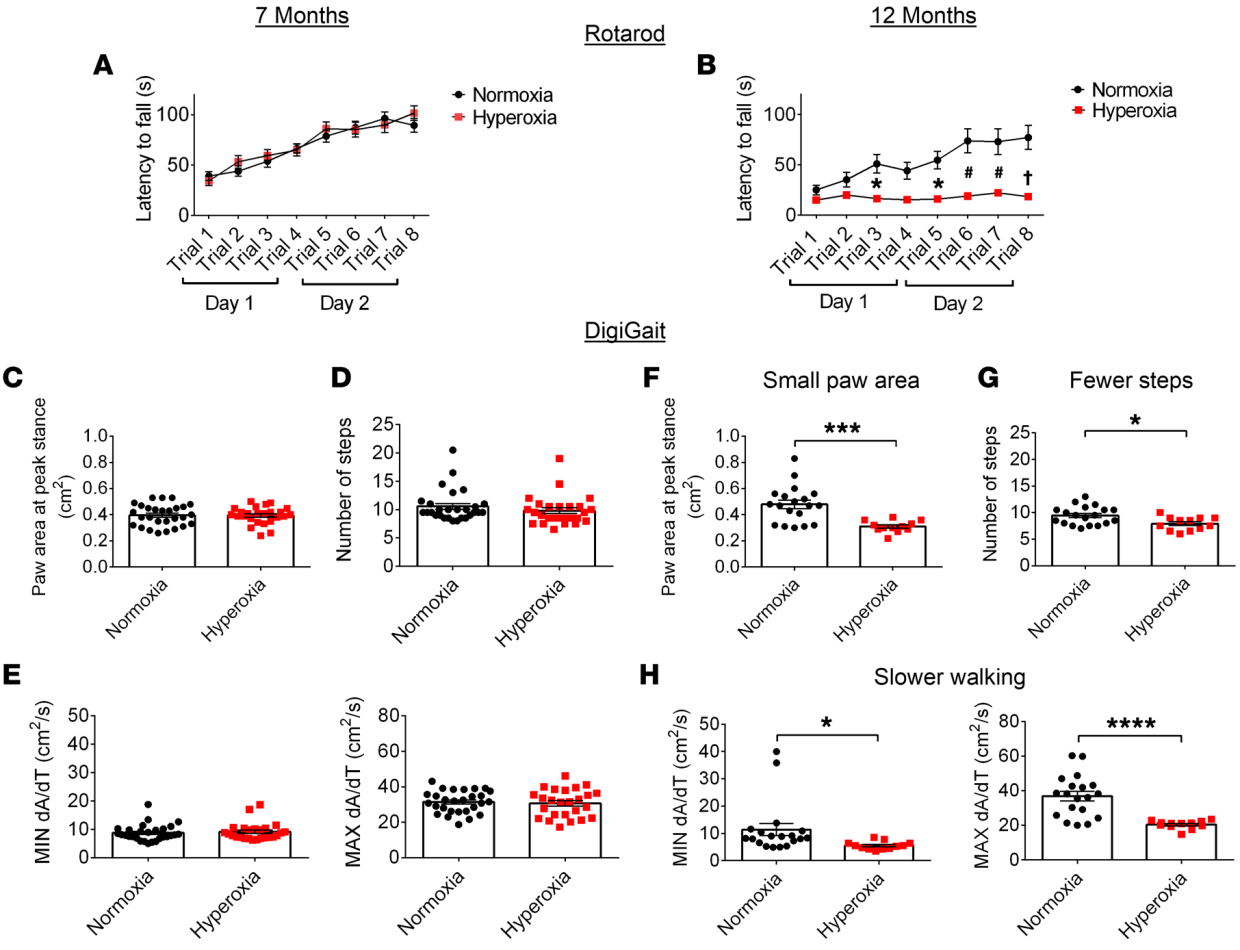
Motor decline with age as a result of developmental hyperoxia exposure. (**A** and **B**) Latency to fall(s) on the rotarod test for 7-month-old mice (**A**, normoxia, *n* = 28; hyperoxia, *n* = 25) and 12-month-old mice (**B**, normoxia, *n* = 19; hyperoxia, *n* = 12). *P < 0.05, ^#^*P* < 0.001; ^†^*P* < 0.0001. Two-way ANOVA with Šidák’s post hoc test for group comparisons. (**C**–**E**) Quantification of DigiGait outcomes for 7-month-old mice (normoxia, *n* = 28, hyperoxia, *n* = 25; unpaired Student’s *t* test), including paw area (cm^2^) at peak stance (**C**), number of steps on the treadmill (**D**), and maximal rate of change in paw area during the propulsion phase (MIN dA/dT) and braking phase (MAX dA/dT) of walking (**E**). (**F**–**H**) Quantification of DigiGait outcome measures for 12-month-old mice (normoxia, *n* = 19, hyperoxia, *n* = 12; *P < 0.05; ***P < 0.001; ****P < 0.0001; unpaired Student’s *t* test). Data are represented as mean ± SEM.

While abnormal neurodevelopmental outcomes of preterm birth have been well studied in childhood and early adulthood (69–71), long-term follow-up to middle age has rarely been investigated. Here, we aimed to determine whether neurodevelopmental outcomes commonly reported in this BPD patient population occur in our BPD mouse model and whether these outcomes change with aging. We used multiple well-described behavioral tests (72) to assess motor and cognitive abilities at 7 and 12 months of age. We assessed motor learning and coordination on the rotarod test and found no differences between the groups at 7 months of age (Figure 15A). In contrast, 12-month-old normoxia-exposed mice were able to stay on the rod significantly longer than hyperoxia-exposed mice (*P* < 0.0001) (Figure 15B). Given that the 12-month-old mice had profound deficits on the rotarod, we conducted a detailed analysis of motor skills by recording mouse gait when walking on an inclined treadmill. At 7 months, no differences were found between the groups (Figure 15, C–E). At 12 months of age, the hyperoxia-exposed mice placed a smaller area of their paws on the treadmill, took fewer steps, and walked more slowly compared with control mice on the treadmill (Figure 15, F–H). These are common observations in human preterm infants who later develop motor impairments (73, 74). Considering the changes in gait, we wondered whether these motor deficits would alter home cage activity when mice were placed into a novel cage. At 7 months, mice from both groups had a similar amount of activity within the cage (Supplemental Figure 7A); however, there was a trend that 12-month-old hyperoxia-exposed mice showed less activity (Supplemental Figure 7B). Overall, for 3 different tasks, the motor phenotypes did not appear until 12 months, which suggests that in mice exposed to hyperoxia during the neonatal period, there was a faster decline in motor abilities as the mice aged.

**Figure 16:**
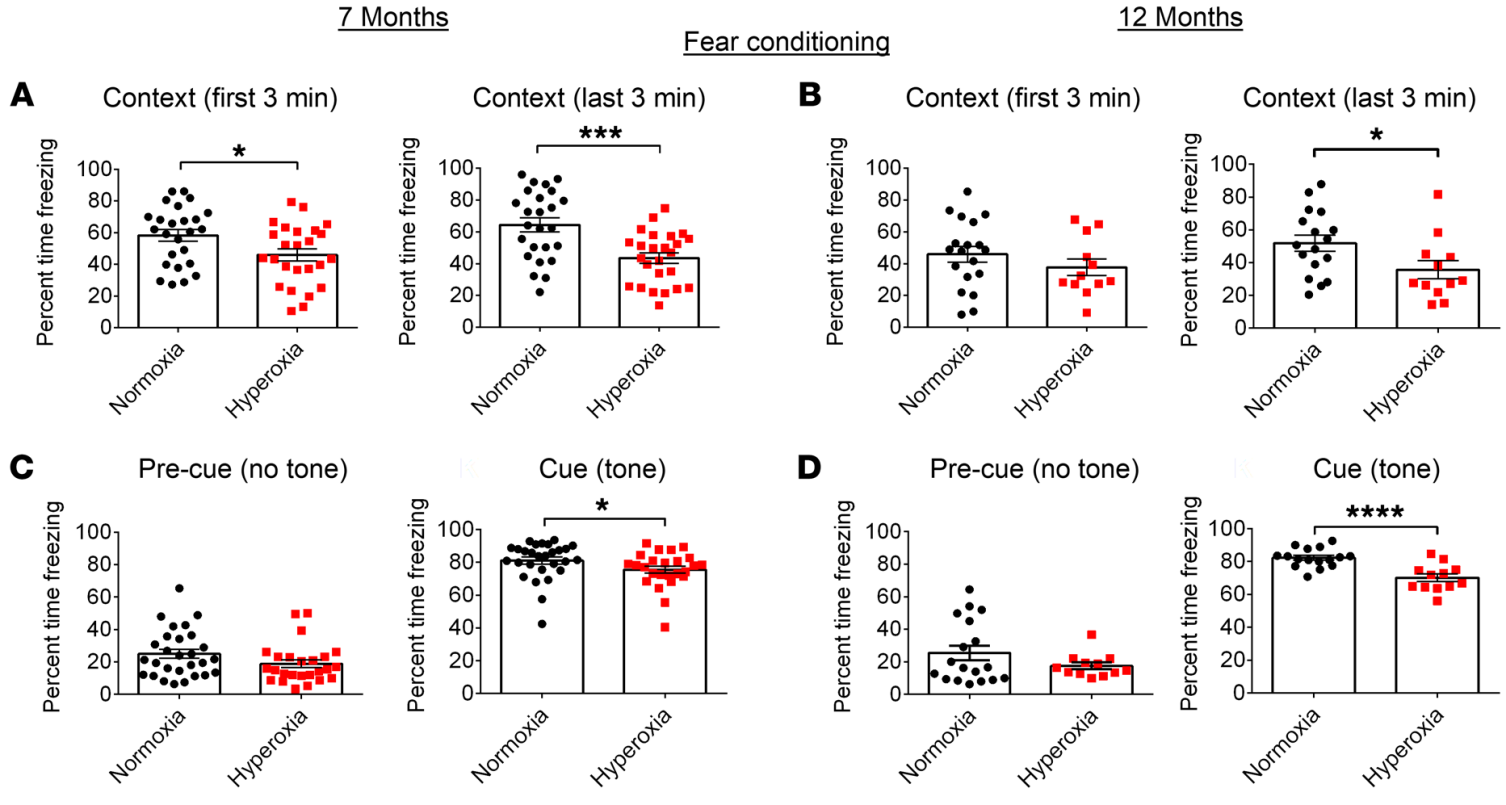
Long-term cognitive deficits following developmental hyperoxia exposure. (**A** and **B**) Quantification of the context fear conditioning task for 7-month-old mice (**A**, normoxia/hyperoxia, *n* = 25) and 12-month-old mice (**B**, context first 3 minutes, normoxia, *n* = 19; hyperoxia, *n* = 12; context last 3 minutes, normoxia, *n* = 17; hyperoxia, *n* = 12). (**C** and **D**) Quantification of the auditory tone fear conditioning task of 7-month-old mice (**C**, normoxia, *n* = 28, hyperoxia, *n* = 25) and 12-month-old mice (**D**, pre-cue [no tone], normoxia, *n* = 19; hyperoxia, *n* = 12; cue [tone], normoxia, *n* = 17; hyperoxia, *n* = 12). *P < 0.05, ***P < 0.001; ****P < 0.0001; unpaired Student’s *t* test. Data are represented as mean ± SEM.

In adulthood, the formation of new neurons within the DG can promote learning and memory (36, 75). This led us to reason that the impaired NPC function following early life hyperoxia in mice may be associated with deficits in learning and memory (76). To test this, we conducted behavioral tests known to assess these specific cognitive abilities, including spatial learning and memory, using the Morris water maze (MWM) task and associative learning using the fear conditioning test (72). Hyperoxia-exposed mice were completely unable to learn the MWM (Supplemental Figure 8, A–D). The distinction between groups was so striking that it was suggested that the mice may have visual deficits. Therefore, we conducted electroretinography (ERG) to assess retinal function. The retinal cells of hyperoxia-exposed mice showed no excitation to light, indicating blindness. Normoxia-exposed animals displayed normal retinal function (Supplemental Figure 9, A and B). Although blindness is a confounding factor on the MWM test, we were able to further investigate learning and memory using the fear conditioning tests. The fear conditioning test trains mice to associate a cue (context or tone) with a shock. If the mice learn to make this association, they will freeze when exposed to the cue (72). During fear conditioning training, there were no significant differences between groups in baseline freezing prior to pairing the shock with the cue and context. In the context test, during the first and last 3 minutes of exposure to the context, the 7-month-old hyperoxia-exposed mice froze less frequently compared with the control mice (Figure 16A), suggesting deficits in hippocampal-dependent associative learning. In the 12-month-old animals, there was a reduction in freezing in the last 3 minutes, but not the first 3 minutes of exposure to context (Figure 16B). This could be due to relatively lower levels of freezing in the older mice, creating a possible floor effect. On the cue test, which is more dependent on the hippocampus and amygdala, the hyperoxia- compared with normoxia-exposed mice at both ages had a significant decline in cue recall (Figure 16, C and D). Thus overall, the fear conditioning test showed that hyperoxia-exposed mice have learning and memory deficits that occur at both 7 months and 12 months of age, which correlates with the hyperoxia-associated reduced hippocampal size, impairments in NPC self-renewal and neurogenesis, and neurovascular deficits. Given that supplemental oxygen is a treatment routinely used for respiratory distress of extremely preterm infants, these findings have major implications for the long-term safety of oxygen administration for neonates.

## Discussion

Our study reveals that early life hyperoxia exposure leads to long-term impairments in cerebrovascular function, neurogenesis, and cognitive deficits and also provides insights for an underlying mechanism contributing to BPD-associated neurodevelopmental deficits.

We found that hyperoxia exposure during early development increased baseline CBF and led to neurovascular uncoupling as well as to higher oxygen concentrations and oxidative stress in the brain. Under these conditions, we showed dramatic vascular remodeling in the cerebral cortex and DG. We found that the expression of key genes involved in proliferation and angiogenesis was dysregulated in neurogenic niche regions directly following postnatal hyperoxia exposure. Furthermore, at 12 months of age, the neurogenic niche regions of hyperoxia-exposed mice showed perturbations of pathways involved in neurotransmission, vascular autoregulation, and neuronal development. Using murine and baboon cells, we also demonstrated that a reduction in the NPC population was associated with impaired NPC self-renewal. Finally, we found that the NPC functional impairments correlated with motor and cognitive deficits during adulthood. These results strongly suggest that NPCs and their vascular niche play a crucial role for appropriate brain development and that impairment due to hyperoxia exposure leads to abnormal brain development and behavioral deficits.

The hyperoxia model mimicking preterm birth–associated brain injury has widespread clinical implications. For example, one hypothesis linking BPD to abnormal neurodevelopment is that the lung injury causes global hypoxia and subsequent brain damage. However, we provide strong evidence that hyperoxia-exposed mice, even following exercise, maintained their arterial oxygen saturation levels within a normal physiological range. This critical observation indicates that in experimental BPD, the lungs and the brain are directly affected by hyperoxia. Therefore, our data support the notion that therapies directly targeted to NPCs and the developing brain vasculature are of immense clinical value. A limitation of our study is that it does not incorporate all preterm birth–associated noxious stimuli, such as ante- and postnatal inflammation, sedation, and steroids (3–5, 77). However, this can also be considered a strength of the model’s focus on supplemental oxygen, providing a detailed examination of the effects of hyperoxia on lung and brain development without these confounding factors.

A major question that arises from our model is the following: how can an excess of oxygen reach and damage the brain? There are several possible contributors. First, one of the most striking results from our study is that early developmental hyperoxia impaired neurovascular function and that this injury was sustained into adulthood. The hyperoxia-induced increased baseline CBF fits well with human data, showing that preterm infants cannot autoregulate their vasculature in response to oxygen treatment (78). Second, in hyperoxia, an increased oxygen tension gradient between the blood and tissue can lead to tissue hyperoxygenation (59). Together, these potential contributors can lead to hyperoxic brain tissue, which coincides with our finding that brain regions of hyperoxia-exposed mice were becoming progressively hyperoxic and showed an increase in oxidative stress over time. A probable source of this progressive hyperoxia is the neurovascular uncoupling observed during development and in adults. Due to the sustained impairment in NVC, an improper amount of oxygen may be delivered to the brain throughout life.

One likely cause of the neurovascular uncoupling is oxygen-induced damage to the vascular structure within the brain, since the integrity of cerebrovascular networks is crucial for proper autoregulation (79). In fact, delayed vascular growth in mouse models of neurodevelopmental disorders, such as autism spectrum disorder, has been previously associated with neurovascular uncoupling in adult mice (56). We found delayed vascular growth in the cerebral cortex and DG. To the best of our knowledge, this is a novel observation in experimental BPD and was further supported by damage to another organ of the central nervous system: the retina. Retinopathy of prematurity (ROP) is a hyperoxia-induced disease, leading to vascular remodeling within the eyes (80).

Interestingly, at 10 months of age, although there was a reduction in vascular density in hyperoxia-exposed mice, there was no change in baseline CBF. Most likely, a homeostatic state developed over time to compensate for the change in vessel structure. However, once the hyperoxia-exposed mice were challenged physiologically with increased stimulation, necessitating an increased demand for blood flow, the reduced vessel density impaired their ability to provide the required blood flow supply.

We also performed gene expression profiling of neurogenic niche regions at P14, during an important developmental window in neurovascular development (81), and found that in hyperoxia-exposed mice there was a significant increase in *Ctla2a* expression, a strong inhibitor of angiogenesis (*P* < 0.01). This finding was further confirmed with FISH analysis. Thus, *Ctla2a* may contribute to the dysregulated vessel growth of hyperoxia-exposed animals. Furthermore, oxygen itself is damaging when administered at high levels, leading to elevated amounts of ROS (82). Normal physiological levels of ROS are required for cell homeostasis (83); however, an excess of ROS within ECs leads to EC dysfunction, apoptosis, and reduced vessel growth (84). OxyBlot analysis revealed that over time, hyperoxia-exposed mice had substantial oxidative stress within their brain tissue, likely contributing to long-term vascular remodelling. Molecular mechanisms that may also contribute to the neurovascular uncoupling of hyperoxia-exposed mice were identified with gene expression profiling of neurogenic niche regions. For example, in the SVZ, prostaglandin-endoperoxide synthase 2 (*Ptgs2*) was downregulated following hyperoxia exposure. *Ptgs2* is the main prostaglandin released during sensory stimulation to cause vasodilation (85). Therefore, the decreased expression of *Ptgs2* may contribute to the impaired ability of vessels to vasodilate to increase blood flow in hyperoxia-exposed animals.

We also demonstrate that early life hyperoxia leads to NPC functional impairments. We show that hyperoxia alone can directly impair NPC self-renewal. Another critical environmental factor that influences NPCs is the surrounding vasculature (86–92). We demonstrate that the reduced vessel formation decreased the amount of anchorage points for NPCs. These anchorage points are crucial for maintaining this NPC population (93, 94). Both factors, (i.e., hyperoxia and vascular impairments) act as an initial insult to NPCs. Impaired NPC self-renewal is associated with a long-term deficiency in neurogenesis as well as learning and memory deficits in adult mice. This is similar to the cognitive impairments associated with preterm birth in humans (69–71). In terms of molecular targets involved in these processes, we showed that neurogenic niche regions from hyperoxia-exposed mice have unique transcriptional signatures compared with the neurogenic niche regions from normoxia-exposed control mice, with more than 100 DEGs in the SVZ and more than 40 DEGs in the hippocampus of 12-month-old mice. GO analysis further revealed the complex hyperoxia-induced injury in these brain regions, as several pathways crucial for normal brain function were perturbed. Thus, there is no single causal mechanism leading to abnormal neurobehavior, but rather many factors, such as oxidative stress, dysregulated vascular autoregulation, inhibited proliferation, and perturbed neuronal differentiation, which collectively play a role in the long-term neurodevelopment deficits.

It is important to note that the initial hyperoxic injury occurred during a critical period of brain development and led to long-term abnormal neurodevelopmental outcomes. Moreover, we report that with age, there is an increase in oxidative stress in neurogenic niche regions and an increase in the number of dysregulated genes in the SVZ and hippocampus, as well as a decline in NPC anchorage points onto blood vessels, the NPC population, newborn neurons, motor learning, and motor coordination abilities. Thus, our data demonstrate that early life insults to the brain vasculature and neurogenic regions lead to a deterioration in brain structures and function, which continues into adulthood. This suggests the importance of an early intervention therapy to mitigate developmental impairments that may have life-long consequences.

Together, these results highlight complex mechanisms that may contribute to preterm birth–associated brain injury. They also demonstrate the need for judicious oxygen administration in the clinic and provide insight into potential therapeutic targets to help mitigate the complications faced by this vulnerable neonatal patient population.

## Methods

### Animal model of hyperoxia-induced lung injury.

Pregnant C57BL/6 mice were shipped from Charles River Laboratories to the University of Ottawa at E13–E16. The pregnant mice were singly housed. The mice were maintained in standard conditions (free access to food and water, 22°C). Mice were housed using a normal 12-hour light/12-hour dark cycle (7 am to 7 pm), except for during the metabolic treadmill test and the behavioral experiments (for which the cycle was reversed for a minimum of 2 weeks prior to the experiments and for the duration of the experiments). Methods were adapted from Alphonse et al. (95) to induce an experimental BPD phenotype. Once the pups were born, the mice were exposed to normoxia (21% O_2_, room air control group) or hyperoxia (85% O_2_, BPD group) using the OxyCycler A84X0V (Biospherix) in Plexiglass chambers (Biospherix) from P0 to P14. Dams were switched every 24 to 48 hours between normoxic and hyperoxic conditions to ensure that the pups received adequate nutrition. Both sexes were assessed in this study. At weaning, mice of the same sex were housed at 2 to 4 mice per cage, unless separated due to fighting. Survival was recorded from P0 to P14 or from P14 to 12 months of age. Body and brain weight were recorded for a subset of P14 and 12-month-old mice at the time of sacrifice (Supplemental Figure 2A).

### Arterial blood oxygen saturation measurements.

Methods were modified from Spitalieri et al. (96). For further details, see Supplemental Methods.

### Lung histopathology and image acquisition.

Lung tissue was collected at P14 and 14 months of age. Adult mice underwent behavioral testing (see *Behavioral experiments* and Supplemental Methods) at 12 months of age. Lungs were processed using modified methods from Li et al. (97) with services from the University of Ottawa Histology Core Facility. For further details, see Supplemental Methods. Lung images were processed using Aperio ImageScope, version 12.3.2.8013, and Fiji (98) (adjustments applied equally to all samples within a comparison).

### Metabolic treadmill test.

Adult mice at 12 to 14 months of age (as described in arterial blood oxygen saturation methods) were tested during the dark cycle and habituated to the dark testing room (red light) for 30 minutes before the treadmill procedure. Each mouse was individually placed into the 1012M-1 Modular Enclosed Metabolic Treadmill for Mice (Columbus Instruments), as adapted by Marcaletti et al. (99). For further details, see Supplemental Methods.

### MRI.

All MRI images were acquired by the University of Ottawa Preclinical Imaging Core Facility (RRID:SCR_021832). For further details, see Supplemental Methods.

### Laser Doppler flowmetry and analysis.

CBF was measured using laser Doppler flowmetry as described previously (55). For further details, see Supplemental Methods.

### Systolic blood pressure.

Mice at 10 months of age were housed in the testing room for a minimum of 1 week prior to training. These mice were previously tested for arterial blood oxygen saturation (see above) at P14. To reduce stress, mice were tested by the same handler at the same time each day. Mice were placed on a BP-2000 Series II Blood Pressure Analysis System (Visitech Systems Inc.) in individual compartments. Tails were confined in tail-cuff holders (100). For further details, see Supplemental Methods.

### Oxygen microsensor measurements and analysis.

Mice at P14 and 10 months of age were anesthetized and mounted according to methods previously described in *Laser Doppler flowmetry and analysis*. These were the same cohorts of adult mice assessed for CBF and allowed a minimum recovery of 1 week before proceeding to oxygen measurements. The oxygen microsensor (OX-10, Unisense A/S) was 2 μm in diameter at the tip, which allowed for a direct, minimally invasive measurement of the oxygen concentration of the brain tissue (101) in the somatosensory cortex, SVZ, and hippocampal regions. Only animals with consistent oxygen concentrations over a period of 2 minutes were used in the analysis.

### OxyBlot Protein Oxidation Detection Kit and analysis.

To assess oxidative stress in the SVZ and hippocampal tissue of mice at P14 and 12 months of age, protein carbonyls were quantified using the OxyBlot assay (MilliporeSigma, S7150) (102), according to the manufacturer’s instructions. Briefly, following tissue homogenization using a mortar and pestle, 20 μg of protein per sample was derivatized and loaded on 10% sodium dodecyl sulfate gels, separated at 150 V for 75 minutes, and transferred to a nitrocellulose membrane. Blocking was performed at room temperature for 1 hour using 1% bovine serum albumin in PBS with Tween 20. Membranes were then incubated with the provided antibodies diluted in blocking buffer (1:150 primary rabbit anti-2,4-dinitrophenyl [DNP] for 1 hour at room temperature; 1:300 horseradish peroxidase–conjugated secondary goat anti-rabbit for 1 hour at room temperature). The membrane was then incubated in Immobilon Crescendo Western horseradish peroxidase substrate for 30 seconds, imaged using Bio-Rad ChemiDoc, and quantified using Image Lab software, version 6.1. Background subtraction was applied equally to all samples within a gel. All adjusted densitometry values were normalized to corresponding Ponceau S loading control values to obtain final protein carbonyl levels. Exclusion criteria included densitometry values that were statistically significant outliers according to Grubb’s test (103). Grubb’s test allowed for the identification of an extreme variant in the data set. Removal of the variant did not change the significance of the unpaired Student’s *t* test (103).

### Cerebral vasculature processing and imaging.

At P14 and 14 to 16 months of age, brain tissue was collected from normoxia- and hyperoxia-exposed mice. Adult mice had undergone behavioral testing (see behavioral methods) at 12 months of age. Brains were sagittally cut in half, immersion fixed in 4% paraformaldehyde for 24 hours at 4°C, and embedded in agarose gel. Using a vibratome, brains were sagitally cut into 120 μm sections, as previously described (104). Sections were then processed, incubated with an antibody against CD31 (1:200; BD Biosciences — Pharmingen, catalog 553370), and mounted onto slides, as previously described (56). 3D 50 μm *Z*-stack images were acquired (×10 magnification) using a Zeiss Axio Imager.M2 with an ApoTome.2 system of 3 fields of view for each of the represented cortical layers (II/III, IV, and V). The average value of the 3 layers (Supplemental Figure 4) was calculated and displayed. Representative images were 3D modeled (105, 106) using the Surfaces module of Imaris, version 9.3 (Bitplane Inc.).

### Cerebrovascular image analysis.

All image analysis was conducted while researchers were blinded to the experimental groups. Computational analysis using Python 2.7 was adapted from protocols previously described (104). Briefly, the following modules were used: Numpy, Scipy, Matplotlib, Opencv2, Igraph, and Scikit-Image. These allowed for immunofluorescent images to be segmented, skeletonized, and quantified for vessel length and branching points.

### Code availability.

The final versions of custom scripts for blood vessel quantification, written in Python, are available on GitHub (https://github.com/chcomin/Neonatal-hyperoxia; branch main, commit ID 3d273e7).

### NPC niche region processing and imaging.

Brain tissue was collected from mice at P14 and 12 months of age. Adult mice had previously been assessed for CBF and oxygen microsensor measurements (see above) at 10 months of age. Brains were cut sagitally in half and the side that was not assessed during the CBF and oxygen measurements was immersion fixed overnight at 4°C in 4% paraformaldehyde. Brains were embedded in agarose and cut coronally into 60 μm thick, serial free-floating sections, as modified according to previously described methods (104). Sections were incubated for 2 hours in blocking solution (1× PBS, 10% donkey serum, 0.5% Triton X-100, 0.5% fish gelatin). To visualize NPCs and the surrounding vasculature, starting from the first serial section, every fourth section throughout the SVZ and DG was incubated in PBT solution (1× PBS with 0.5% Triton X-100) with antibodies against nestin (1:500; R&D Systems, catalog AF2736), Sox2 (1:500; Abcam, catalog ab97959), and CD31 (1:200) overnight at room temperature with gentle shaking. The following day, sections were washed 4 times (5 minutes each) with PBT solution. This was followed by incubation with secondary antibodies (all Invitrogen; 1:300, donkey α-rat Alexa Fluor 488, catalog A-21208; 1:1,000, donkey α-rabbit Alexa Fluor 555, catalog A-32794; 1:1,000, donkey α-goat Alexa Fluor 647, catalog A-32849), as well as counterstaining with DAPI (Invitrogen, D1306) for 3 hours at room temperature with gentle shaking. To visualize newborn neurons, starting from the second serial section, every fourth section throughout the SVZ and DG was processed and incubated as described above, with antibodies against DCX (1:500; Santa Cruz Biotechnology Inc., catalog SC-8066; secondary antibody, 1:1,000; Invitrogen, donkey α-goat Alexa Fluor 647, catalog A-32849), and also counterstained with DAPI. Sections were washed twice with PBT solution, followed by 2 washes in phosphate buffer solution to remove the salt and Triton X-100. Sections were then mounted onto slides using Fluoromount-G (Fisher Scientific, 5025973). 3D 20 μm *Z*-stack images were acquired (×20 magnification) using a Zeiss LSM800 AxioObserver Z1 confocal microscope using ZEN 2.6 (blue edition). For further details, see Supplemental Methods.

### Gene expression profiling of SVZ and hippocampal tissue.

RNA was isolated from the SVZ and hippocampus of P14 and 12-month-old mice using the E.Z.N.A. HP Total RNA Kit (Omega Bio-tek) per the manufacturer’s instructions. Génome Québec Innovation Centre (Montreal, Canada) conducted the Clariom S Assay mouse microarray (Thermo Fisher Scientific, 902930). Transcriptome Analysis Console software, version 4.0.2.15 (Thermo Fisher Scientific), was used to perform DEG analysis, applying thresholds of a gene-level fold change of more than 2 or less than –2 and gene level *P* value of less than 0.05, as assessed by the empirical Bayes statistical test. Microarray information was deposited on ArrayExpress (accession E-MTAB-11332). GO analysis was conducted using R 4.1.0 with the clusterProfiler package (107).

### FISH analysis.

Methods were adapted from Bordeleau et al. (108). For further details, see Supplemental Methods.

### NPC subpopulation processing and imaging.

The SVZ tissue was processed for both P14 and 12-month-old mice as described above (104). To identify the different subpopulations of NPCs, the following primary antibodies were used: Sox2 (1:400 Novus Biologicals, catalog AF2018), Tbr2 (1:200 Abcam, catalog AB23345), and Ki67 (1:500 Thermo Fisher Scientific, catalog 14-5698-82), followed by secondary antibodies (all Invitrogen at 1:1,000: donkey α-rat Alexa Fluor 488, catalog A-21208; 1:1000, donkey α-rabbit Alexa Fluor 555, catalog A-32794; 1:1000, donkey α-goat Alexa Fluor 647, catalog A-32849). All tissue was counterstained with DAPI. 3D 20 μm *Z*-stack images were acquired (×20 magnification) on a Zeiss Axio Imager.M2 with an ApoTome.2 system. *Z*-projections were produced using ZEN 2.6 (blue edition). For further details, see Supplemental Methods.

### Neurosphere assays with murine-derived NPCs.

NPCs from mice at P14 and 14 months of age were isolated and cultured using methods adapted from Fujitani et al. (109). For further details, see Supplemental Methods.

### Baboon NPC isolation and neurosphere assays.

We enrolled 6 neonatal baboons (male and female) in our study, housed at the Texas Biomedical Research Institute, San Antonio, Texas, USA. Three baboons were term animals, delivered vaginally. NPCs were isolated from these animals at gestational days 185, 190, and 194. Three baboons were preterm, delivered via C-section. NPCs were isolated from these animals at gestational days 141, 127, and 125. Brain tissue was collected and stored in artificial cerebral spinal fluid on ice. The subependyma of the lateral ventricles and the DG of the hippocampus were dissected, digested with papain, and mechanically dissociated. Cells were filtered through a 40 μm mesh before being plated at a density of 37,500 cells/mL in DMEM/F-12 media containing 20 ng/mL FGF (MilliporeSigma, F0291), 20 ng/mL EGF (MilliporeSigma, E9644), 8 μg/mL heparin, 2% B-27, 1% N-2 (Thermo Fisher Scientific, 17502048), and 1% antibiotic-antimycotic. Cells isolated from preterm baboons were cultured overnight and then exposed to 60% O_2_ for 48 hours. Cells were then transferred to 21% O_2_. Control term baboon cells remained in 21% O_2_ for the duration of the experiment. Primary neurospheres were counted and imaged once the majority of neurospheres cultured at 21% O_2_ (controls) reached a minimum diameter of 50 μm. Neurospheres were dissociated with TrypLE Express Enzyme, washed in DMEM/F-12, and plated in media described above at a density of 3,750 cells/mL. Secondary neurospheres were counted and imaged once the majority of neurospheres cultured at 21% O_2_ (controls) reached a minimum diameter of approximately 50 μm. Images of neurospheres were acquired (×10 magnification) using ToupView 3.7 (ToupTek) on an Olympus IX 50 inverted system microscope.

### Behavioral experiments.

Behavioral experiments and analysis were conducted by the staff of the University of Ottawa Behavior and Physiology Core Facility per previously established protocols (72). For further details, see Supplemental Methods.

### ERG.

Retinal function was assessed at 6, 9, and 15 to 17 months of age. Animals at 9 months of age had previously been tested for arterial blood oxygen saturation at P14, and animals at 15 to 17 months of age had previously been tested for behavior at 7 months of age (see above). Mice were weighed and habituated to the dark testing room overnight prior to the ERG. ERGs were conducted on a CELERIS system (Diagnosys LLC) with Espion software, version 6.61.12 (Diagnosys LLC) using methods modified from Wassmer et al. (110). For further details, see Supplemental Methods.

### Statistics.

Statistical analyses were conducted using GraphPad Prism, version 6.0, software or Transcriptome Analysis Console software, version 4.0.2.15. All data are represented as mean ± SEM. For analyses conducted using GraphPad Prism, unpaired, 2-tailed Student’s *t* test was used to compare 2 groups and 1-way ANOVA with post hoc Tukey’s test or 2-way ANOVA with post hoc Šidák’s test was used to compare multiple groups. *P* < 0.05 was considered statistically significant. For analyses conducted using Transcriptome Analysis Console software, an empirical Bayes statistical test was used. DEG analysis applied thresholds of a gene-level fold change of more than 2 or less than –2 and gene level *P* value of less than 0.05. Statistical details of all experiments can be found in the figure legends. If applicable for the experiment, exclusion criteria are described in the respective methods.

### Study approval.

All mouse procedures were approved by the University of Ottawa Animal Care Committee. All nonhuman primate baboon procedures were approved by the University of Texas Health Science Center at San Antonio Institutional Animal Care and Use Committee.

## Author contributions

MAL designed experiments, established the animal model, assisted with and analyzed the oxygen saturation, CBF, and oxygen concentration experiments, conducted blood pressure measurements, acquired and analyzed lung and brain images, performed neurosphere assays, assisted with the analysis of the metabolic treadmill, behavioral, and visual experiments, analyzed the OxyBlot assays, analyzed the microarray DEG data and conducted the GO analysis, interpreted data, and wrote the manuscript. XT performed the CBF and oxygen concentration experiments. SZ and LX assisted with lung processing, mouse neurosphere assays, and RNA isolation for the microarray. SBM contributed intellectual guidance and assisted with baboon neurosphere assays. JO immunostained and imaged type B neural stem cells and type C NPCs. MFA conducted the FISH immunostaining and imaged brain regions. CHC conducted vascular quantification of the cortical brain regions. HAB and ANB conducted and analyzed vision function tests. YS cryosectioned brain tissue for FISH analysis. MW conducted microarray DEG analysis. AGM contributed material and intellectual guidance. CLB assisted with the collection of baboon tissue and contributed intellectual guidance. AV contributed intellectual guidance, assisted with establishing the animal model, and conducted the OxyBlot assays with SZ. CT supervised the vision function tests. SRS contributed intellectual guidance and assisted with the collection of baboon tissue. RSS, DCL, JW, and BL contributed intellectual guidance, provided training and material, and edited the manuscript. BT supervised the study, contributed intellectual guidance, provided financial support, and edited the manuscript.

## Supplementary Material

Supplemental data

## Version 1 09/22/2022

In-Press Preview

## Version 2 11/15/2022

Electronic publication
