# Supplementary material for: Neonatal hyperoxia in mice triggers long-term cognitive deficits via impairments in cerebrovascular function and neurogenesis
Source: J Clin Invest. 2022 Nov 15;132(22):e146095. doi: 10.1172/JCI146095 (PMC9663164; doi:10.1172/JCI146095)
Supplement: Supplemental data [file jci-132-146095-s175.pdf]

Supplemental Methods

Arterial blood oxygen saturation measurements. The oxygen saturation measurements were conducted at P14 and at 12-14-months of age. Adult mice had undergone behavioral testing (see behavioral methods) at 7 months of age. Both age groups were tested at rest; however, adult mice were also assessed immediately following treadmill exercise (see treadmill methods). Mice were anesthetized with isoflurane and the Mouse Ox Plus® oximeter sensor (Starr Life Sciences Corp.) was placed over the shaved, right thigh of each mouse to detect blood oxygen saturation of the femoral artery. Arterial blood oxygen saturation, heart rate (beats per minute), and respiratory rate (breaths per minute) were recorded for a minimum of 25 seconds using the Mouse Ox Plus® basic software.

Lung histopathology and image acquisition. Lungs were intratracheally inflation fixed with 10% buffered formalin, under 20 cm H_2O pressure, for 5 minutes. Lungs were immersion fixed in 10% buffered formalin for 48 hours at room temperature and immersed in 70% ethanol for 24 hours at room temperature. Lungs were then paraffin-embedded, cut into 4 µm sections, and stained with hematoxylin and eosin at the University of Ottawa Histology Core Facility. To visualize lung structure, images (x20 magnification) were acquired on an Aperio CS2 slide scanner (Leica) using the Aperio eSlide Manager (Leica).

Metabolic treadmill test. The treadmill speed increased as follows: 0 cm/s for 5 minutes, 8cm/s for 5 min, 15cm/s for 5 min, 20 cm/s for 5 min, 25 cm/s for 5 min, then increased by 3 cm/s every 2 minutes. Mice remained on the treadmill until exhaustion, as determined by the mouse remaining on the shock bar (~0.5mA) for 10 seconds, the mouse being unable to run, or if the respiratory exchange ratio became greater than or equal to 1 or dramatically increased within 4-6 minutes and then reached a plateau. Oxygen consumption and carbon dioxide release were measured every 30

seconds using the Oxymax®/ comprehensive lab animal monitoring system (CLAMS) and software (Columbus Instruments).

MRI. Mice were 14 months of age at time of testing and had previously been assessed for behavior at 12 months of age (see behavior methods). MRI was conducted using a 7 Tesla General Electric/Agilent MR901 machine. Coronal, 2D fast spin echo T_2-weighted brain images were acquired (TE = 25 ms, TR = 6000 ms, ETL = 8, bandwidth = 15.6 KHz, FOV = 2.5 cm, slice thickness = 0.5 mm, matrix = 265 x 256, scan time = 7 min). The size of the whole brain, lateral ventricles, and hippocampal regions were measured using Fiji. The total area of anatomically comparable sections was measured using the polygon tool and was multiplied by the thickness of each MRI slice.

Laser doppler flowmetry and analysis. The same cohorts of animals were tested at P14 and 10 months of age. Briefly, once the animal was mounted in the stereotactic equipment and the skull was exposed, the flowmeter (BLF22, single-channel tissue perfusion monitor, Transonic Systems Inc.) was placed above the left somatosensory cortex. Baseline CBF measurements were recorded. To assess neurovascular coupling, whiskers on the right side of the animal were stimulated for 20 second intervals a minimum of 3 times. An average of three stimuli was used for the baseline and whisker stimulated CBF values for each animal.

Systolic blood pressure. Systolic blood pressure was measured 5 times, to ensure consistency, followed by 10 trials which were recorded. Training occurred over 5 consecutive days. After a 2-day interval, systolic blood pressure was measured on the acclimatized mice over 5 consecutive days, following the same procedure as during the training sessions. Only consistent measurements of at least 8 of the 10 trials, per day, were used in the analysis. The average systolic blood pressure of approximately 50 trials per mouse was calculated.

NPC niche region image analysis. All image analysis was conducted while blinded to the experimental groups. 3D modelling of z-stack images was conducted using the surfaces module of Imaris 9.3 to measure the volume of i) the SVZ and ii) the SGZ (for NPC quantification) or DG (for newborn neuron quantification) of sections. For P14 mice, a total sum of 2 sections per region, per animal was used in the analysis. For 12-month-old mice, a total sum of 3 sections per region, per animal was used in the analysis. For NPC quantification, computational analysis of the SGZ was conducted with Imaris 9.3 (Bitplane Inc.) using the spots module to quantify NPCs ($Sox2^+$, $nestin^+$) and confirmed with manual inspection. For the SVZ, images were manually quantified using the cell counter module of Fiji. The contact points of $CD31^+$ ECs with nestin processes of NPCs in the DG was quantified manually using the cell counter module of Fiji. Newborn neurons (DCX^+) were manually quantified in the SVZ and DG using the cell counter module of Fiji. Images were processed in Fiji (adjustments applied equally to all images within a comparison) and are displayed as maximum intensity Z-projections.

FISH analysis. Mouse brains were fixed overnight at 4°C in 4% paraformaldehyde. Brains were rinsed in PBS and cryopreserved in 20% sucrose. Samples were then collected as 15 µm thick coronal sections and stored at -80°C until further processing. Brain sections were prepared for RNAScope assay following the manufacturer's instructions (Advanced Cell Diagnostics, 320535 Rev A). Briefly, antigen retrieval was conducted at 100°C for 5 minutes in Target Retrieval solution (Advanced Cell Diagnostics, 322000). Sections were then incubated in Protease III digestion solution (Advanced Cell Diagnostics, 322340) for 30 minutes at 40°C. Labeling of target RNA, *Mm-Ctla2a*, was conducted by following the manufacturer's instructions (Advanced Cell Diagnostics, document number 320293). The probe was constructed by the manufacturer and

provided in the RNAscope® Fluorescent Multiplex Detection Reagents Kit (Advanced Cell Diagnostics, 320851). After labeling and amplification, the slides were washed four times, for 5 minutes, in Wash Buffer (Advanced Cell Diagnostics, 310091). Slides were then rinsed in distilled water and mounted with Fluoromount-G or ProLongGold with DAPI. Sections were imaged as 8 µm z-stacks and were acquired (x20 magnification) on a Zeiss Axio Imager.M2 with an ApoTome.2 system. Images were manually quantified using the cell counter module of Fiji. Images were processed in Fiji (adjustments applied equally to all images within a comparison) and are displayed as a maximum intensity Z-projection. A total sum of two sections per region per animal was used in the analysis. A minimum of 200 cells/ section from 3 fields of view were counted. The cells expressing 2 or more identifiers of the RNA of interest, *Ctla2a*, were counted as positive. A percentage of *Ctla2a* positive cells was calculated from the total cells counted.

NPC subpopulation image analysis. Images were manually quantified using the cell counter module of Fiji. The area of each counted region was measured using the polygon tool of Fiji. Images were processed in Fiji (adjustments applied equally to all images within a comparison) and are displayed as a maximum intensity Z-projection.

Neurosphere assays with murine-derived NPCs. Adult mice had previously been assessed for behavior at 12 months of age (see behavior methods). Briefly, the subependyma of the lateral ventricles was removed, digested with papain (Worthington, PAPL LS003118), and mechanically dissociated. Cells were filtered through a 40 µm mesh (Corning, 352340) and plated for a primary neurosphere assay at a cell density of 10 cells/ µL in Dulbecco's Modified Eagle Medium/Nutrient Mixture F-12 (DMEM/F-12) media (Thermo Fisher Scientific, 11330057) containing 20 ng/mL fibroblast growth factor-2 (FGF) (Sigma, F0291), 20ng/mL epidermal growth factor (EGF) (Sigma, E1257), 2 µg/mL heparin (Sigma, H3149), 2% B-27 (Life Technologies, 10889-038), and

1% antibiotic-antimycotic (Thermo Fisher Scientific, 15240-062). Primary neurospheres were counted and imaged 7-8 days after plating. Neurospheres were then dissociated with TrypLE™ Express Enzyme (Thermo Fisher Scientific, 12605-028), washed in DMEM/F-12, and plated in media (described above) at a density of 2 cells/µL. Secondary neurospheres were counted and imaged 7-8 days after plating. Images were acquired (x20 magnification) on a Nikon Eclipse TE2000-E brightfield microscope using NIS-Elements AR 3.0 (Nikon) and all images were processed in Fiji (adjustments applied equally to all images within a comparison).

Behavioral experiments. Adult mice at 7 months and 12 months of age were handled for 2 days before the experiments commenced and the person conducting the testing was blinded to the treatment groups. All testing was conducted during the dark cycle and in red light, with the mice being habituated to the red-light testing room for ~30-60 minutes prior to testing, unless otherwise indicated.

Rotarod. Mice had four trials (10-minute inter-trial interval (ITI)) over a period of 2 consecutive days on an accelerating rotarod (IITC Life Science Inc.). For each trial, the rod was set to accelerate from 4 to 45 rpm in 300 seconds, followed by 300 seconds at 45 rpm.

DigiGait™. Mouse gait was recorded for a minimum of 3 seconds on the transparent DigiGait™ treadmill (Mouse Specifics, Inc.) that was set to a speed of 18 cm/sec, and an incline of 8 degrees. Videos were blindly analyzed using DigiGait™ Analysis software (Mouse Specifics, Inc.).

Home cage locomotor activity. Mice were placed individually into clean housing cages which were then placed for 4 hours into the Home Cage Locomotor Activity Infrared Beam Break frames that were paired with the Fusion software (Omnitech Electronics, Inc.).

Morris water maze. Each mouse was habituated to the testing room in 140 lux light for 30 minutes. A single black X (2.8 cm thick, 15cm long x 13.5cm wide) was placed on the back wall of the

room as a cue. Each mouse was placed in a circular pool, measuring 132 cm in diameter, filled with water colored white with tempera paint maintained at 23°C. The pool contained a hidden platform (10 cm in diameter) in one of its 4 quadrants. Each mouse was trained to find the platform for four trials each day (ITI of 30 minutes) for nine days total. During each trial, the mouse had 1 minute to find the platform. On the 10th day, the platform was removed, and the mouse was given one minute to search for the platform. Each trial was tracked and analyzed using Ethovision software (Noldus). If the mouse did not find the platform within 1 minute, the program automatically stopped.

Fear conditioning. Mice were not habituated to the testing room prior to the experiment and the experiment was performed using the PhenoTyper® boxes (Noldus) with grid shock floors (Med Associates). The overhead room lights were on and no lights were projected by the boxes. On the training day, mice were placed into the box. The mice remained in the box for 2 minutes, after which a 30 second 80 dB tone played, followed by a 2 second 0.45mA foot shock. This tone-shock pairing was repeated two times with a 1-minute interval. The final tone-shock pair was followed by a 30-second interval. The mice were then returned to their home cages. On day 2, to assess contextual memory, mice were placed in the same box for the same duration as training with no tone or shock. On day 3, to assess cued memory, mice were placed in a different Phenotyper® box from the one used for training and the contextual memory test. This box was altered through the addition of a plastic floor, triangular plastic walls, and a vanilla scent. The room was lit with red light and the boxes were lit with both white and yellow light simultaneously. The mice were in the modified box for six minutes with the 80 dB tone playing during the last three minutes, in the absence of any shock. All trials were recorded and freezing behavior was scored using Ethovision software (Noldus). Mice that froze less than 10 seconds were not included in the analysis.

Electroretinography. Animals were anesthetized with an intraperitoneal injection of ketamine hydrochloride (50 mg kg^{-1}; Narketan®) and medetomidine hydrochloride (1 mg kg^{-1}; Cepetor®). Anesthesia was maintained throughout the test and the medetomidine was reversed after 1 hour using atipamezole hydrochloride (1 mg kg^{-1}; Antisedan®). Eyes were dilated for 10 minutes prior to ERG testing with one drop each of 1% tropicamide (Mydriacyl, Alcon) and 2.5% phenylephrine hydrochloride (Mydfrin, Alcon). A topical anesthetic (0.5% proparacaine hydrochloride; Alcain, Alcon) was applied to each eye. 1 mL of saline was administered subcutaneously, prior to testing. Ag/AgCl contact stimulators were placed on both corneas in combination with Optixcare Veterinary Eye Lube (Aventix) to ensure stimulator contact and corneal hydration. A gold loop reference electrode was placed on the tongue, a needle electrode was placed sub-dermally in the head, and a grounding needle electrode was placed subcutaneously in the tail. Retinal function was assessed with the following 3 protocols. The simultaneous ERG and visually evoked potentials (VEP) protocol stimulates the retina with a blue and green coloured flash at an intensity of 0.05 candela seconds (cd.s)/m^2 and a frequency of 1 Hz. The c-wave protocol stimulates the retina with a white-6500K flash at an intensity of 150 cd.s/m^2 and a frequency of 1 Hz. The photopic negative response (PhNR) protocol stimulates the retina with a white-6500K flash at an intensity of 20 cd.s/m^2 and a frequency of 2 Hz.

Fundus photography. Mice were anesthetized, dilated, and maintained as described in the ERG protocol above. Fundus imaging was acquired using Streampix 3 (Norpix) on a Micron III microscope (Phoenix Technology Group) to inspect retinal morphology.

Supplemental Data

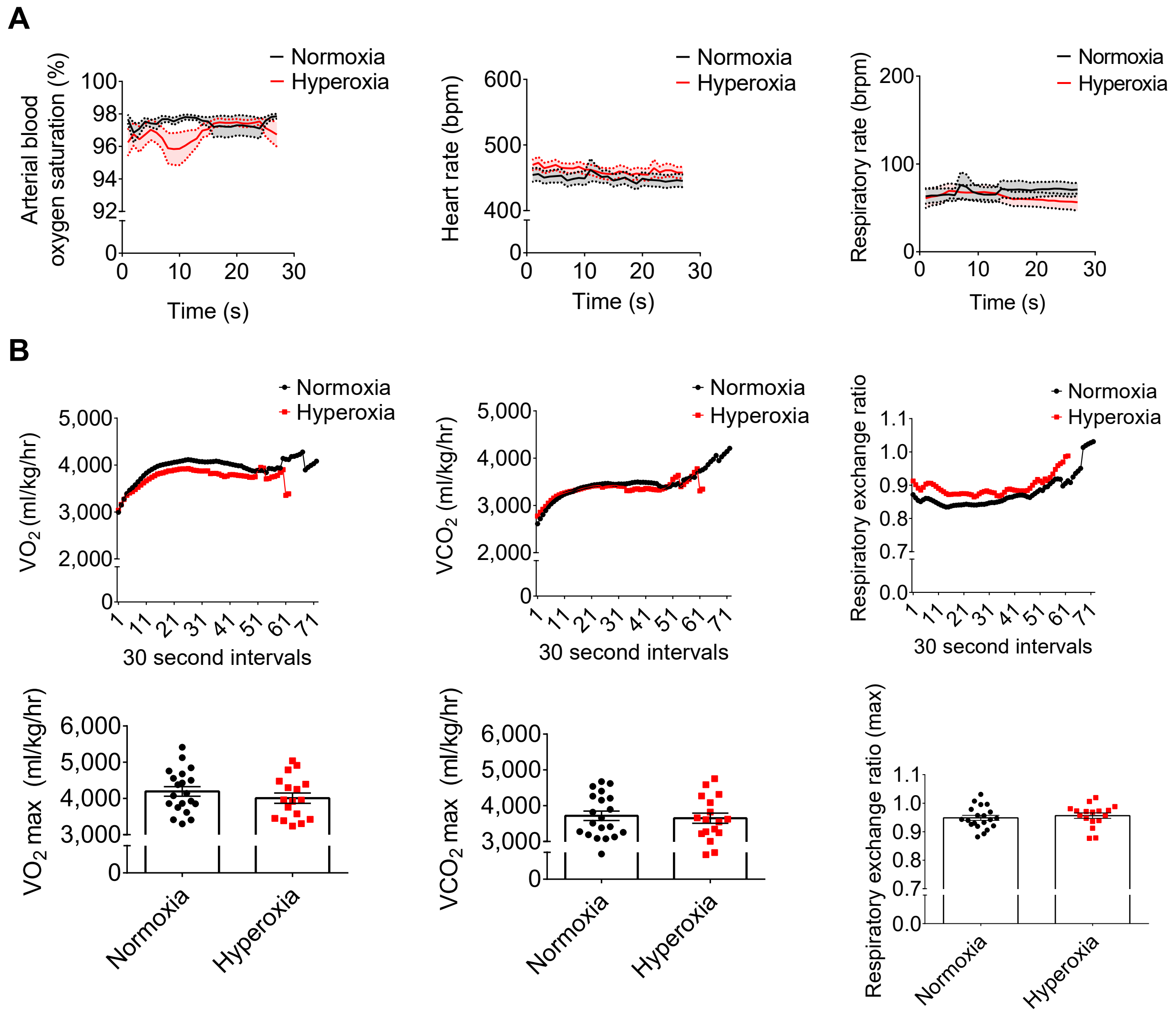

Supplemental Figure 1. Early life hyperoxia does not influence arterial oxygen saturation, oxygen consumption, or carbon dioxide production in adulthood. (**A**) Three outcome measures were assessed for 12-14-month-old mice at rest. From left to right: blood oxygen saturation of the femoral artery; heart rate (beats per minute, bpm); and respiratory rate (breaths per minute, brpm) (normoxia, n=20; hyperoxia, n=17; two-way ANOVA with Sidak post hoc test for group comparisons). (**B**) Volume of oxygen consumption, volume of carbon dioxide release, and the respiratory exchange ratio (VCO_2 / VO_2) for the total duration of metabolic treadmill testing (top) and at maximum capacity (bottom) (normoxia, n=20; hyperoxia, n=17; two-way ANOVA with Sidak post hoc test for multiple comparisons and unpaired Student's t test, respectively). Mice were tested at 12-14 months of age. Data are expressed as mean ± SEM.

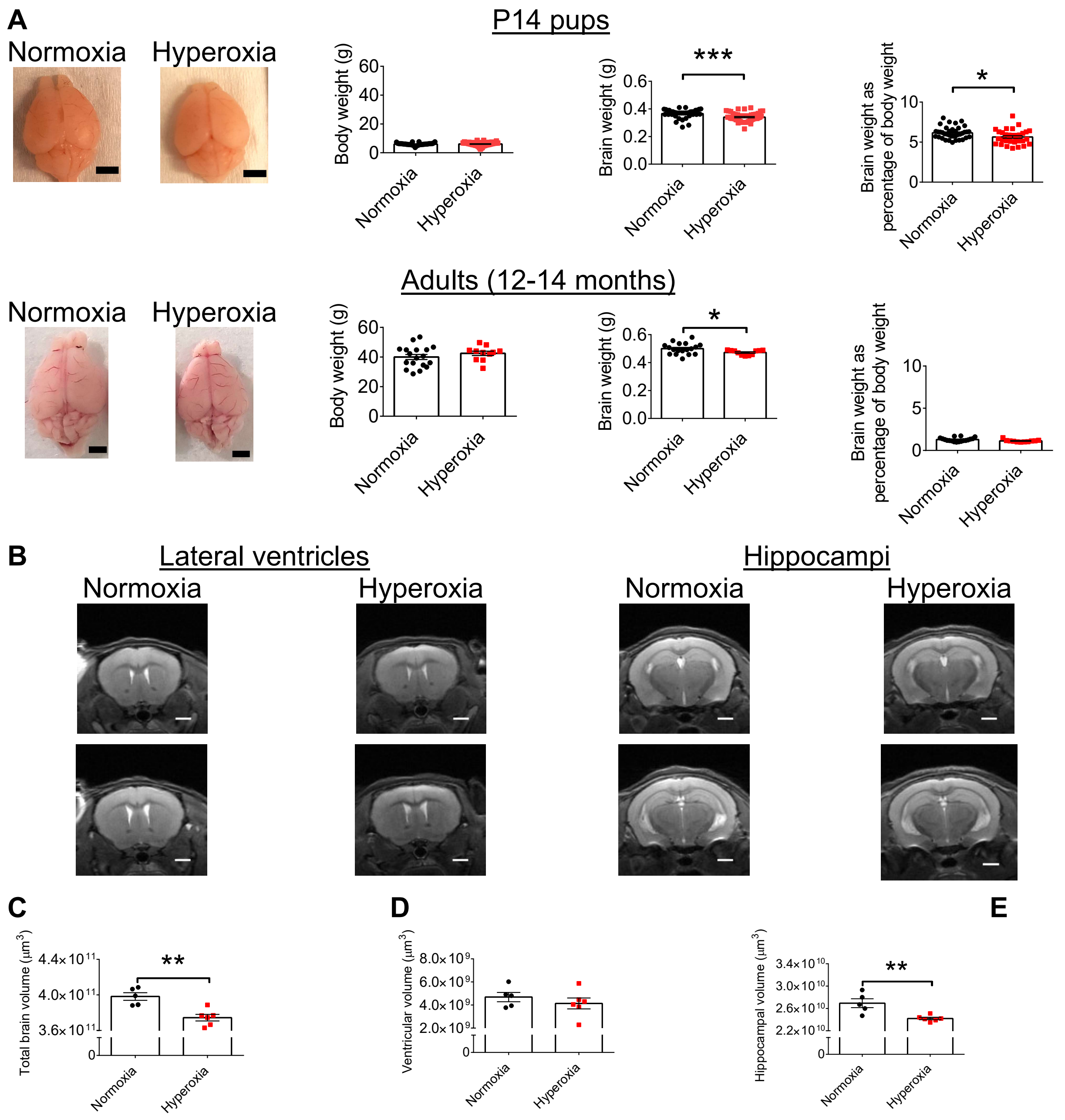

Supplemental Figure 2. Delayed brain growth after early developmental hyperoxia exposure. (**A**) Representative images (left) of brains from normoxia and hyperoxia-exposed mice at P14 and 12 months, scale bar, 3mm. Body weight (g), brain weight (g), and brain weight as a percentage of body weight (right) for P14 (top, normoxia, n= 38, hyperoxia, n=48) and 12-14-month-old mice (bottom, normoxia, n= 18, hyperoxia, n=11); $*P < 0.05$, $***P < 0.001$; unpaired Student's t test. (**B**) Representative MRI images of the ventricular (left) and hippocampal (right) regions of a normoxia and hyperoxia-exposed mouse. Scale bar, 1500 µm. (**C**) Quantification of the total brain volume (normoxia, n=5; hyperoxia, n=6; $**P < 0.01$; unpaired Student's t test). (**D**) Quantification of the volume of the lateral ventricles (normoxia, n=5; hyperoxia, n=6; unpaired Student's t test). (**E**) Quantification of the volume of the hippocampal regions (normoxia, n=5; hyperoxia, n=6; $**P < 0.01$; unpaired Student's t test). Data are expressed as mean ± SEM.

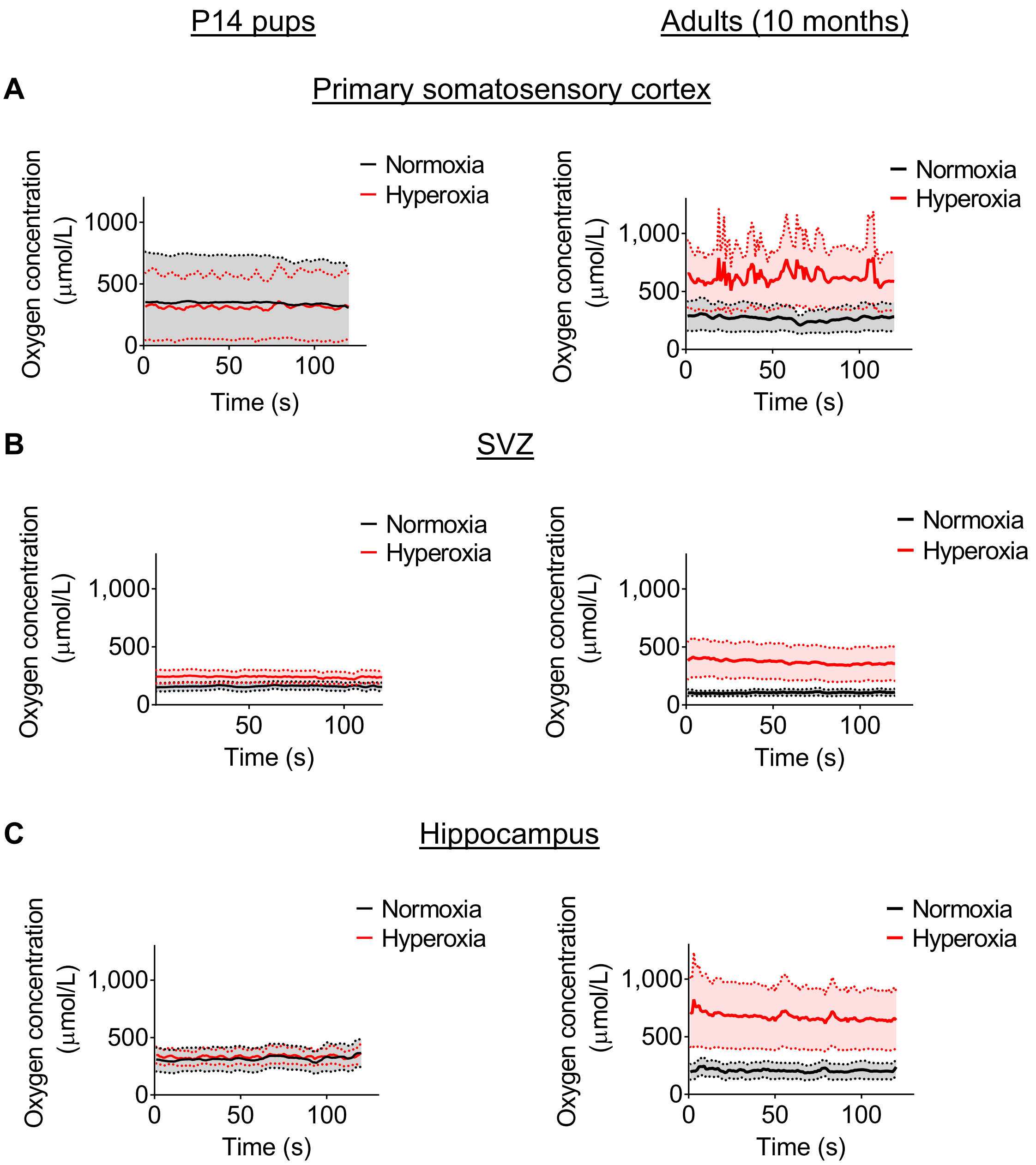

Supplemental Figure 3. Brain regions of hyperoxia-exposed mice show trend of becoming progressively hyperoxic during aging. (**A**) Oxygen concentration (µmol/L) of the primary somatosensory cortex in anesthetized P14 (left) and 10-month-old (right) mice (P14, normoxia, n=8; hyperoxia, n=9; 10-month-old, normoxia, n=12; hyperoxia, n=11). Two-way ANOVA with Sidak post hoc test for multiple comparisons. (**B**) Oxygen concentration (µmol/L) of the subventricular zone (SVZ) region in anesthetized P14 (left) and 10-month-old (right) mice (P14, normoxia, n=6; hyperoxia, n=9; 10-month-old, normoxia, n=10; hyperoxia, n=11). Two-way ANOVA with Sidak post hoc test for multiple comparisons. (**C**) Oxygen concentration (µmol/L) of the hippocampal region in anesthetized P14 (left) and 10-month-old (right) mice (P14, normoxia, n=9; hyperoxia, n=8; 10-month-old, normoxia, n=11; hyperoxia, n=10). Two-way ANOVA with Sidak post hoc test for multiple comparisons. Data are expressed as mean ± SEM.

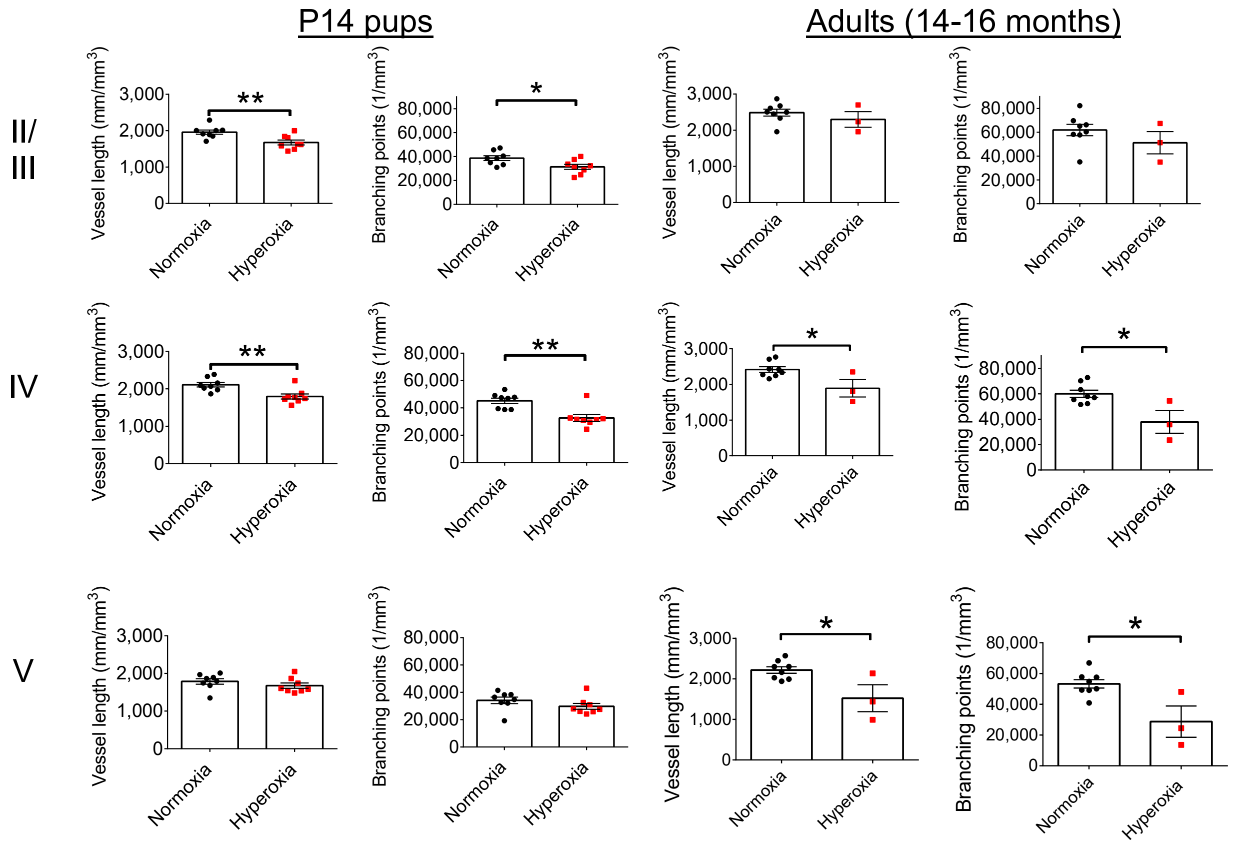

Supplemental Figure 4. Vascular remodelling in multiple neocortical layers after early life hyperoxia exposure. Vessel length and number of branching points in neocortical layers II/III, IV, and V for P14 and 14-16-month-old mice (P14 mice, normoxia, n=8, hyperoxia, n=8; adult mice, normoxia, n=8, hyperoxia, n=3; *$P < 0.05$, **$P < 0.01$; unpaired Student's t test). Data are expressed as mean ± SEM.

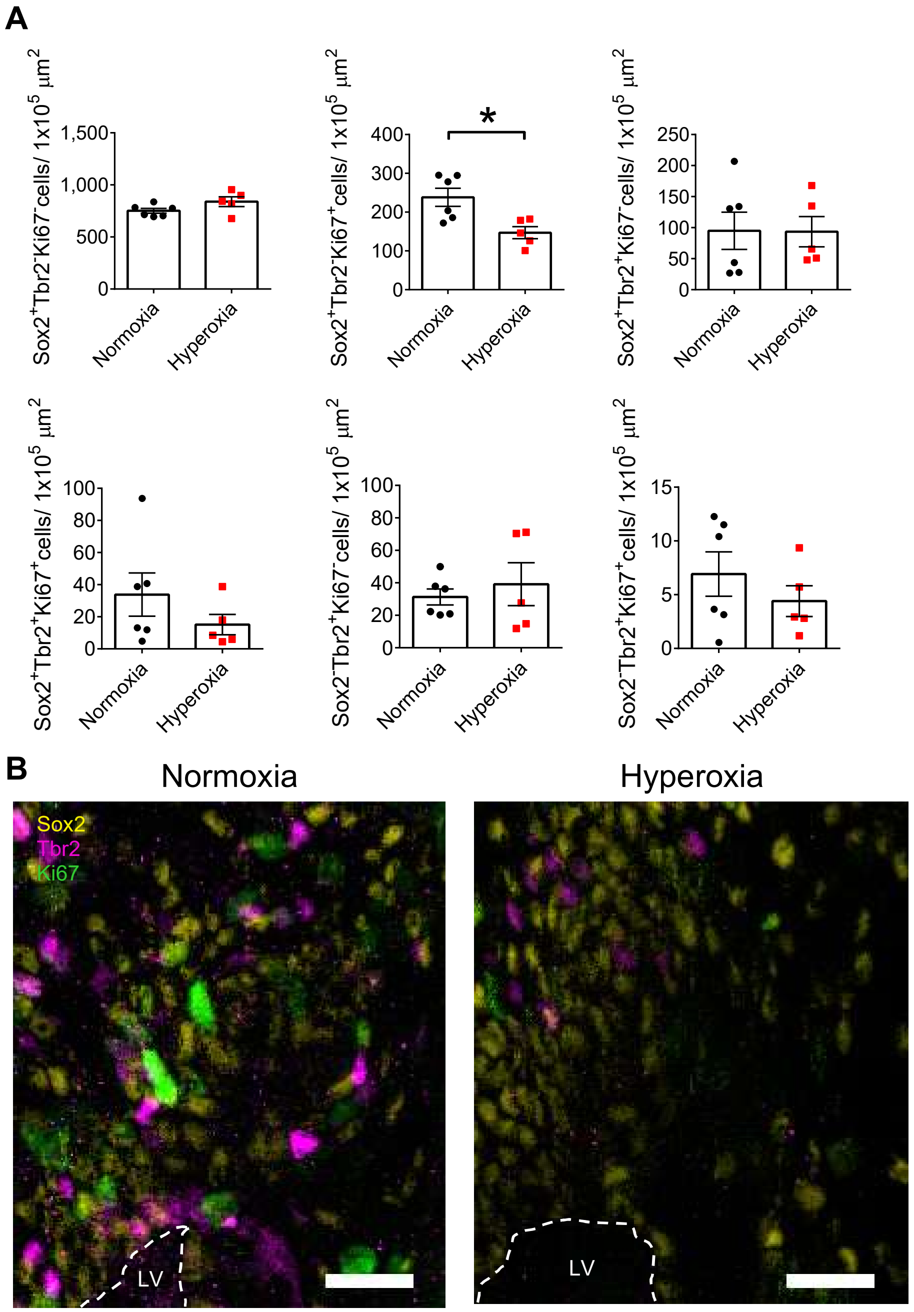

Supplemental Figure 5. Hyperoxia exposure in early life leads to a significant reduction in the proliferating neural stem cell population. (A) Quantification of type B neural stem cells (Sox2^{+}Tbr2^{-}Ki67^{-}), proliferating type B neural stem cells (Sox2^{+}Tbr2^{-}Ki67^{+}), immature type C NPCs (Sox2^{+}Tbr2^{+}Ki67^{-}), proliferating immature type C NPCs (Sox2^{+}Tbr2^{+}Ki67^{+}), mature type C NPCs (Sox2^{-}Tbr2^{+}Ki67^{-}), and proliferating mature type C NPCs (Sox2^{-}Tbr2^{+}Ki67^{+}) in the SVZ of P14 mice (normoxia, n=6; hyperoxia n=5; *$P < 0.05$; unpaired Student's t test). (B) Representative images of the SVZ region of normoxia vs. hyperoxia-exposed mice. Scale bar, 20 μm. Data are expressed as mean ± SEM. LV, lateral ventricle.

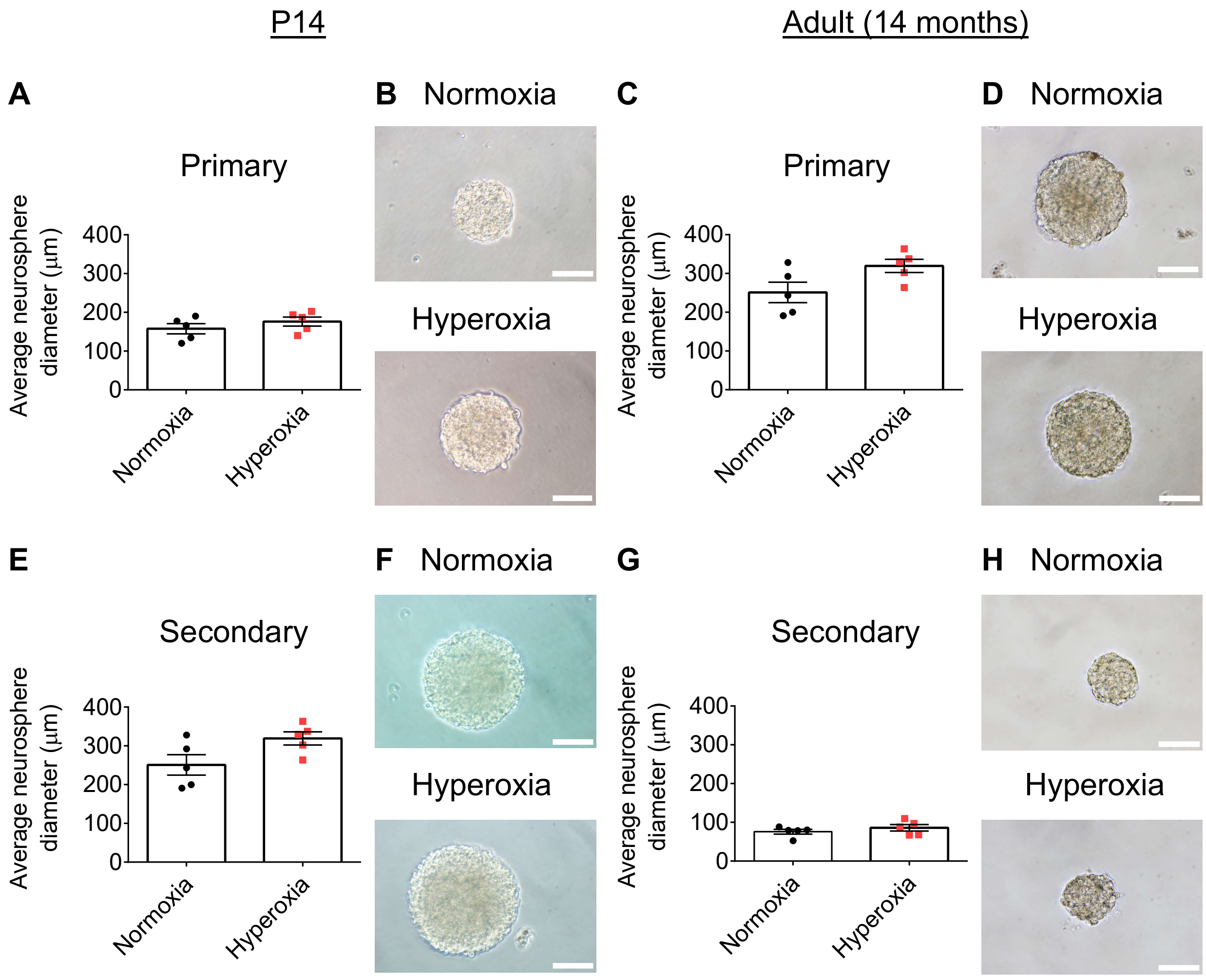

Supplemental Figure 6. NPCs from mice exposed to hyperoxia form neurospheres of similar size compared to those from normoxia mice. (**A** and **B**) Quantification (A) and representative images (B) of the average primary neurosphere diameter formed by NPCs from P14 mice. (**C** and **D**) Quantification (C) and representative images (D) of the average primary neurosphere diameter formed by NPCs from 14-month-old mice. (**E** and **F**) Quantification (E) and representative images (F) of the average secondary neurosphere diameter formed by NPCs from P14 mice. (**G** and **H**) Quantification (G) and representative images (H) of the average secondary neurosphere diameter formed by NPCs from 14-month-old mice. Normoxia, n=5; hyperoxia, n=5; unpaired Student's t test. Data are expressed as mean ± SEM. Scale bar, 100 µm.

Home-cage assessment of movement

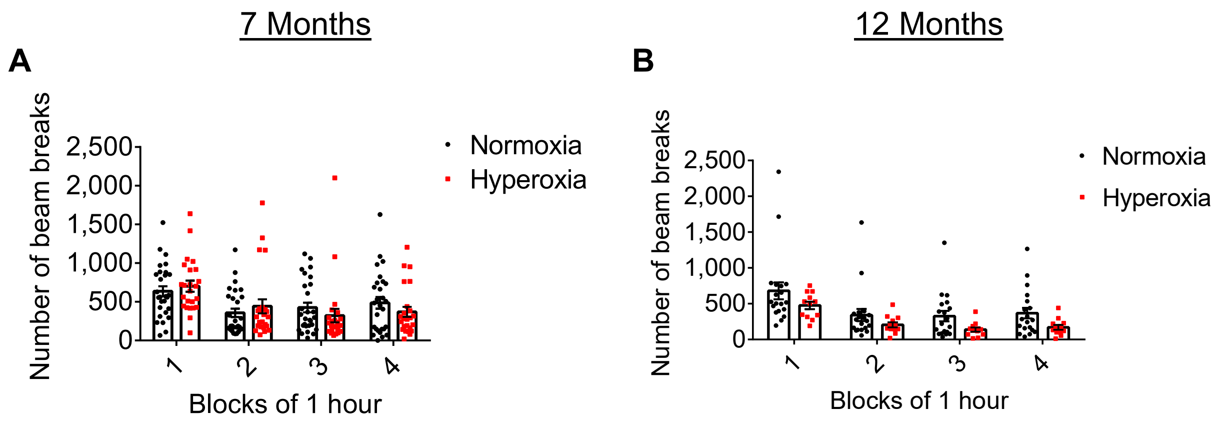

Supplemental Figure 7. Hyperoxia exposed mice show a trend of less movement at 12 months of age compared to normoxia exposed mice. (**A** and **B**) Distance travelled (cm) during the home-cage assessment of movement for 7-month-old mice (A, normoxia, n=28; hyperoxia, n=24) and 12-month-old mice (B, normoxia, n=19; hyperoxia, n=12). Two-way ANOVA with Sidak post hoc test for group comparisons. Data are expressed as mean ± SEM.

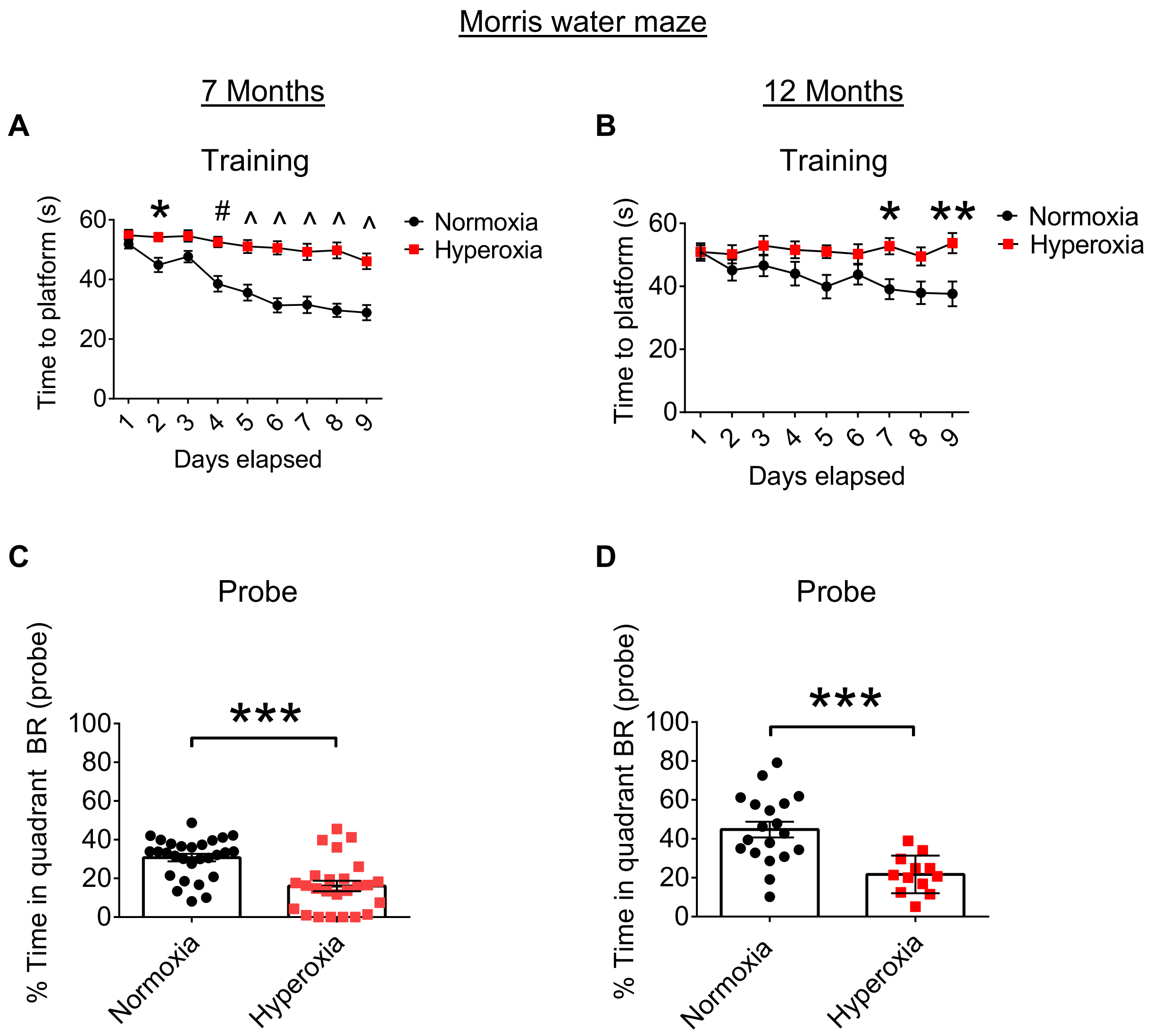

Supplemental Figure 8. Hyperoxia-exposed mice perform poorly on the Morris Water Maze (MWM) learning and memory assessment. (**A** and **B**) Amount of time for mice to reach the platform (s) during MWM training at 7 months (A, normoxia, n=28; hyperoxia, n=25) and 12 months (B, normoxia, n=19; hyperoxia, n=12). Two-way ANOVA with Sidak post hoc test for multiple comparisons. (**C** and **D**) Percent time that mice spent in the correct quadrant on the MWM test (probe) day at 7 months (C, normoxia, n=28; hyperoxia, n=25) and 12 months (D, normoxia, n=19; hyperoxia, n=12). *$P < 0.05$; ** $P < 0.01$; ***$P < 0.001$; #$P < 0.001$; ^$P < 0.0001$. Data are expressed as mean ± SEM.

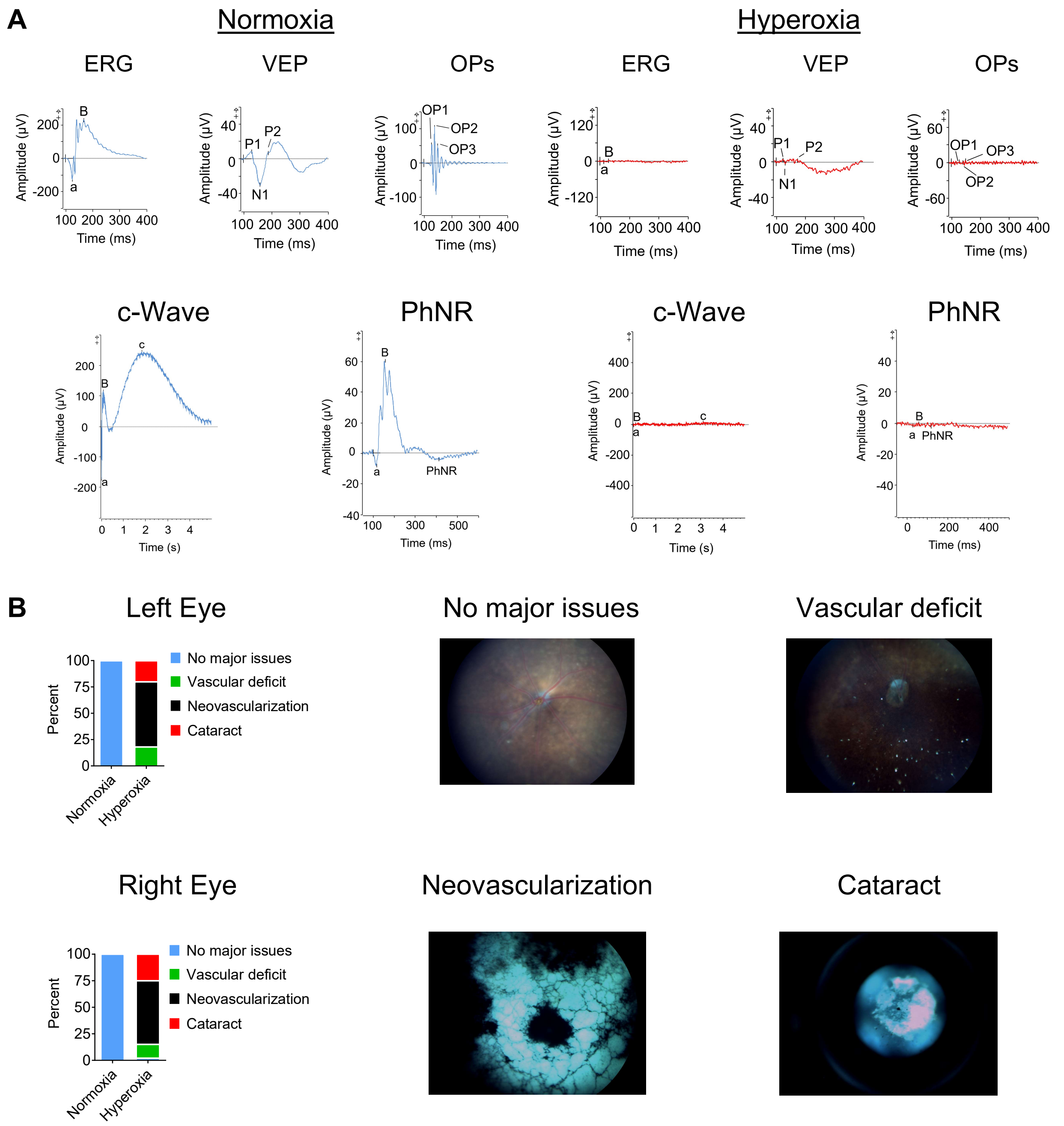

Supplemental Figure 9. Hyperoxia exposure causes major ocular damage to the retinal vasculature, leading to blindness. (**A**) Representative electrophysiological traces of retinas from normoxia (left) vs. hyperoxia (right)-exposed mice. ERG, electroretinography; VEP, visually evoked potentials; OPs, oscillatory potentials; PhNR, photopic negative response; P, positive; N, negative. Each wave represents the excitation of specific retinal cells: a-wave, cones and rods; b-wave, muller glia, bipolar cells; c-wave, retinal pigmented epithelium; VEPs, visually evoked signal in the visual cortex; OPs, amacrine cells; PhNR, retinal ganglion cells. (**B**) Quantification (%) of the number of mice with no major ocular issues, vascular deficits, neovascularization, and cataracts (left) of mice at 6, 9, and 15-17 months of age (normoxia, n=40; hyperoxia, n=45). Representative fundus images of retinas (right).

Supplemental Table 1. Antibody Information.

Antibody	Vendor	Catalogue Number	Dilution
Rat anti-CD31	BD Pharmingen	553370	1:200
Goat anti-Doublecortin	Santa Cruz	sc-8066	1:500
Rat anti-Ki67	Thermo Fisher Scientific	14-5698-82	1:500
Goat anti-Nestin	R&D Systems	AF2736	1:500
Rabbit anti-Sox2	Abcam	ab97959	1:500
Goat anti-Sox2	Novus Biologicals	AF2018	1:400
Rabbit anti-Tbr2	Abcam	ab23345	1:200
Rabbit anti-DNP	MilliporeSigma	S7150 (Included in OxyBlot Kit)	1:150
Donkey α-Rat IgG (H+L) Highly Cross-Adsorbed Secondary Antibody, Alexa Fluor 488	Invitrogen	A-21208	1:300
Donkey anti-Rabbit IgG (H+L) Highly Cross-Adsorbed Secondary Antibody, Alexa Fluor Plus 555	Invitrogen	A-32794	1:1000
Donkey anti-Goat IgG (H+L) Highly Cross-Adsorbed Secondary Antibody, Alexa Fluor Plus 647	Invitrogen	A-32849	1:1000
HRP goat anti-rabbit	MilliporeSigma	S7150 (Included in OxyBlot Kit)	1:300

Supplemental Table 2. P14 SVZ characterized differentially expressed genes (DEGs) with associated neural functions.

DEG	Expression Change (hyperoxia vs. normoxia)	Function	Study
Ctla2a	Increase	Inhibitor of angiogenesis	Maruyama et al., 2021 (1)
Mrgprb2	Decrease	Receptor specific to mast cells, activation leads to increase in pro-inflammatory cytokines and recruitment of immune cells	Green et al., 2019 (2)
Cd93	Increase	Negative regulator of astrogenesis for neural stem cells (NSCs); role in the regulation of inflammation; associated with phagocytotic activity of microglia	Liang et al., 2020 (3); Griffiths et al., 2018 (4); Maas et al., 2020 (5)
Nr4a3	Increase	Transcriptional target of p53; inhibits proliferation; induces apoptosis	Fedorova et al., 2019 (6)
Cplx3	Decrease	Helps facilitate synaptic release of neurotransmitters	Vaithianathan et al. 2015 (7)

Supplemental Table 3. P14 hippocampal characterized DEGs with associated neural functions.

DEG	Expression Change (hyperoxia vs. normoxia)	Function	Study
Cd93	Increase	Negative regulator of astrogenesis for NSCs; role in the regulation of inflammation; associated with phagocytotic activity of microglia	Liang et al., 2020 (3); Griffiths et al., 2018 (4); Maas et al., 2020 (5)
Ctla2a	Increase	Inhibitor of angiogenesis	Maruyama et al., 2021 (1)
Lcn2	Increase	Activates inflammasome and weakens tight junctions of the blood brain barrier	Mondal et al., 2020 (8)
Adm	Increase	Vasodilator; contributor in memory loss with aging	López et al., 2002 (9); Larrayoz et al., 2017 (10)
Ifitm3	Increase	Increased in pro-inflammatory microenvironments. Leads to increase in γ-secretase, which leads	Hur J-Y et al., 2020 (11)

		to increase in amyloid-β (a key contributor to Alzheimer's disease)	
Prnd	Increase	Dpl (protein coded by Prnd) at high levels can be neurotoxic (leading to neurodegeneration and ataxia)	Moore et al., 2001 (12)
Mgp	Increase	Inhibits bone morphogenetic protein	Yao et al., 2013 (13)
Igfbp2	Increase	Important for early brain development, synaptic transmission, and cognition	Khan et al., 2019 (14)
Gadl1	Increase	Decreases expression of genes that play a role in cell migration	Wu et al., 2019 (15)
Kl	Increase	Neuroprotective (antioxidant) effect on neurons of the hippocampus	Zeldich et al., 2014 (16)
Rbm47	Increase	Increased levels prevent cancer progression in brain tissue, while loss increases proliferation of cancerous cells	Vanharanta et al., 2014 (17)
Tlr1	Increase	Increased expression in the brain during inflammation	Mishra et al., 2006 (18)
Cdh4	Decrease	Important for vascular development in the brain	Krishna et al., 2009 (19)
Folr1	Increase	Deficiency leads to neurodegeneration	Steinfeld et al., 2009 (20)
Kcnj13	Increase	Encodes for a potassium channel (not activated by depolarization, i.e., inwardly rectifying); may play a role in homeostasis of neurons and glial cells.	Papanikolaou et al., 2019 (21)

Supplemental Table 4. 12-month SVZ characterized DEGs with associated neural functions.

DEG	Expression Change (hyperoxia vs. normoxia)	Function	Study
Tnfaip6	Increase	Upregulated under pro-inflammatory microenvironments; anti-inflammatory effects	Li et al., 2018 (22)
Crtac1	Decrease	Acts as an antagonist to axon growth inhibitors	Sato et al., 2011 (23)
Dusp16	Increase	Deficiency leads to an expansion of the NPC population and an increase in neurogenesis	Zega et al., 2017 (24)

Ptgs2 (COX-2)	Decrease	Main prostaglandin released during sensory stimulation to cause vasodilation of vessels	Lacroix et al., 2015 (25)
Igfbp6	Decrease	Specifically binds and inhibits IGF2, inhibiting glioma cell proliferation	Oliva et al., 2018 (26)
Pdzrn3	Decrease	Crucial for vessel formation	Sewduth et al., 2014 (27)
Nmbr	Decrease	May play a role in thermal sensitivity	Mishra et al., 2012 (28)
Pvalb	Increase	Pvalb interneurons are inhibitory for synaptic transmission; Decrease in these neurons is associated with neurological disorders	Ruden et al., 2021 (29)
Nr2f2	Increase	Important temporal cue for NPC differentiation; decrease will result in neurogenesis	Naka et al., 2008 (30)
Nr4a2	Decrease	Critical for long-term memory of recognizing objects and locating their position	McNulty et al., 2012 (31)
Cabp1	Decrease	Inhibits synaptic transmission by regulating calcium channels	Li et al., 2013 (32)
Fam81a	Decrease	Potential function in synaptic transmission	Dosemeci et al., 2019 (33)
Hapln4	Increase	Important for extracellular matrix structures around neurons	Edamatsu et al., 2018 (34)
Prkca	Decrease	Increased activity has been associated with Alzheimer's disease	Callender et al., 2017 (35)
Glra1	Increase	Disruption of activity can lead to motor function problems	Schaefer et al., 2012 (36)
Tfap2d	Decrease	Decreases retinal ganglion cell number and projections to the brain	Li et al., 2016 (37)
Igf1	Increase	Disruption of signalling can protect against Alzheimer's disease phenotype	Gontier et al., 2015 (38)
Vamp1	Increase	Association between an increase in expression and Alzheimer's disease risk	Sevlever et al., 2015 (39)
Frzb	Decrease	Can decrease axon growth	Rich et al., 2018 (40)
Slc30a3	Decrease	Critical for zinc allocation to synaptic vesicles	Cole et al., 1999 (41)
Fhl2	Decrease	Decrease in activity may lead to NSC shift from neurogenesis to gliogenesis	Kim et al., 2019 (42)

Neurod2	Decrease	Decreased activity leads to a decrease in "excitability" of pyramidal neurons	Chen et al., 2016 (43)
Slc17a7	Decrease	Gene that encodes for vesicular glutamate transporter 1; crucial for excitatory (glutamatergic) neurotransmission	Wojcik et al., 2004 (44)
Lef1	Increase	May play an important role in neuronal differentiation	Armenteros et al., 2018 (45)
Cox6a2	Increase	Part of cytochrome c oxidase (final enzyme in electron transport chain); deletion leads to oxidative stress in neurons	Sanz-Morello et al., 2020 (46)
Cbln1	Increase	Plays an important role in synapses for aging mice	Seigneur et al., 2018 (47)
Adra1b	Increase	May play a role in memory formation	Perez et al., 2020 (48)
Ndnf	Decrease	Increases neuronal migration and growth of neuronal processes	Kuang et al., 2010 (49)
C1ql3	Decrease	Plays an important role in excitatory synapses	Martinelli et al., 2016 (50)
Kcnh1	Decrease	Encodes for a potassium voltage-gated channel; important for brain activity	Kessi et al., 2020 (51)
Fam19a1	Decrease	Deficiency leads to impaired behavior (motor, memory, & fear acquisition)	Yong et al., 2020 (52)
Rora	Increase	Encodes for a receptor that regulates transcription; decreased levels associated with autism spectrum disorder	Devanna et al., 2014 (53)
Ramp3	Increase	Promotes signalling that increases migration of endothelial cells	Mackie et al., 2019 (54)
Tnc	Increase	Depending on microenvironment, may play a role pro-inflammatory signalling cascades & blood-brain disruption	Okada et al., 2021 (55)
Napepld	Increase	May play an important role in synaptic transmission	Egertová et al., 2008 (56)
Grm1	Increase	Encodes for mGluR1; Important for long-term depression of synaptic transmission	Lüscher et al., 2010 (57)
Agt	Increase	Part of the renin-angiotensin system (RAS); increased activation can lead to vasoconstriction	Nakagawa et al., 2017 (58)

Plekhg1	Increase	Plays a role in reorienting vascular endothelial cells; may lead to increased white matter	Traylor et al., 2019 (59)
Kcng1	Decrease	Encodes for a potassium channel	Cioli et al., 2014 (60)
Kcnc2	Increase	Encodes for potassium channel; increase could lead to hyperexcitability of neurons	Stern et al., 2020 (61)
Htr5b	Increase	Serotonin receptor; Increase associated with symptoms reminiscent of Rhett syndrome in mice	Vogelgesang et al., 2017 (62)
Prkcd	Increase	Role in pro-inflammatory response and blood-brain barrier increased permeability	Tang et al., 2018 (63)
Styx1a	Decrease	High levels associated with increased glioma growth	Tomar et al., 2019 (64)
Kcnv1	Decrease	Encodes for a component of a potassium channel	Hugnot et al., 1996 (65)
Basp1	Decrease	NSC marker	Manganas et al., 2021 (66)
Bmp3	Decrease	May play a role in blood-brain barrier integrity	Morita et al., 2021 (67)
Chrnb3	Increase	Nicotinic-acetylcholine receptor; depleted in Parkinson's disease	Bar-Shira et al., 2014 (68)
Syt17	Decrease	Important for growth of neuronal processes and synaptic transmission	Ruhl et al., 2019 (69)
Syt9	Increase	Important for neurotrophin release	Wang et al., 2016 (70)
Gprin1	Decrease	Important for the growth of neuronal processes	Chen et al., 1999 (71)
Cacna1g	Increase	Encodes for a voltage gated calcium channel; associated with encephalopathy	Berecki et al., 2020 (72)
Mas1	Decrease	Angiotensin receptor; activation leads to vasodilation; may play a role in angiogenesis	Foulquier et al., 2019 (73)
Fhdc1	Increase	May be important for microtubule function	Galbraith et al., 2019 (74)
Chrnb4	Increase	Nicotinic-acetylcholine receptor; Decreased expression associated with social amnesia	Salas et al., 2013 (75)
Tac2	Increase	Upregulation associated with increased aggression and antisocial behavior	Zelikowsky et al., 2018 (76)
Gjc (Connexin45)	Increase	Increases MASH1+ progenitor cell proliferation	Khodosevich et al., 2012 (77)

Prox1	Increase	Important for differentiation and migration of oligodendrocytes to corpus callosum	Bunk et al., 2016 (78)
Cdkn1a (p21)	Decrease	Cell cycle inhibitor; reduces proliferation of NSCs	Kippin et al., 2005 (79)
Ntng1	Increase	Important signal to recruit microglia to neuronal axons	Fujita et al., 2020 (80)
Tcf7l2	Increase	Plays an important role in NSC maintenance during development, unclear role in adult tissue	Bem et al., 2019 (81)
Shox2	Increase	Decreased expression leads to impairment in inferior colliculus and cerebellum, ultimately causing motor coordination issues	Rosin et al., 2015 (82)
Grid2ip	Increase	Encodes for Delphilin; actin nucleator in Purkinje neurons	Silkworth et al., 2018 (83)
Htr2a	Decrease	Serotonin receptor; decreased expression due to epigenetic changes is associated with early onset schizophrenia and bipolar disorder	Abdolmaleky et al., 2011 (84)
Tbr1	Decrease	Critical transcription factor for the transition of neural progenitors to postmitotic cortical neurons	Bedogni et al., 2010 (85)
Fmod	Decrease	Promotes glioma cell migration	Mondal et al., 2017 (86)
Kcnj4	Decrease	Potassium channel (not activated by depolarization, i.e., inwardly rectifying); may be important for synaptic transmission	Inanobe et al., 2002 (87)
Eomes (Tbr2)	Decrease	Intermediate neural progenitor marker; increases neurogenesis	Lv et al., 2019 (88)
Slc17a6	Increase	Gene that encodes for vesicular glutamate transporter 2; crucial for excitatory (glutamatergic) neurotransmission	Birgner et al., 2010 (89)

Supplemental Table 5. 12-month hippocampal characterized DEGs with associated neural functions.

DEG	Expression Change (hyperoxia vs. normoxia)	Function	Study
Cdr2	Decrease	Downregulates c-Myc in Purkinje neurons; c-Myc can lead to proliferation or apoptosis	Okano et al., 1999 (90)
Hrh2	Decrease	Encodes for a histamine receptor; deficit impairs object recognition and leads to increased anxiety	Schneider et al., 2014 (91)
Sgms2	Decrease	Important for the cell membrane/ cell membrane proteins (particularly drug transporters)	Zhang et al., 2011 (92)
Otx2	Decrease	Decreased expression decreases adult neurogenesis	Planques et al., 2019 (93)
Prlr	Decrease	Promotes excitation of neurons	Patil et al., 2014 (94)
Elovl7	Decrease	Enzyme that contributes to the elongation of fatty acids; Mutation associated with Parkinson's disease	Deák et al., 2019 (95)
Kcne2	Decrease	Encodes for a voltage-gated potassium channel; reduced expression can decrease neuron excitability	Ying et al., 2012 (96)
Sulf1	Decrease	May be involved in inhibiting the growth of neuronal processes	Joy et al., 2015 (97)
Ttr	Decrease	May have a neuroprotective role against the accumulation of amyloid beta, a main phenotype of Alzheimer's disease	Li et al., 2011 (98)
A2m	Decrease	Increased level associated with Alzheimer's disease	Varma et al., 2017 (99)
Fosb	Decrease	Decreased expression leads to impaired neurogenesis and epilepsy	Yutsudo et al., 2013 (100)
Epn3	Decrease	Increased expression promotes migration of glioblastoma cells	Wang et al., 2018 (101)
Rdh5	Decrease	Mutations associated with blindness	Sergouniotis et al., 2011 (102)
Aqp1	Decrease	Primarily a water channel, but also permeable to oxygen, carbon dioxide, and nitric oxide	Badaut et al., 2014 (103)
Clic6	Decrease	Intracellular chloride channel; may play a role in dopamine signalling	Griffon et al., 2003 (104)

Folr1	Decrease	Plays an important role in folate function (transferring methyl group to molecules, e.g., amino acids); decrease associated with neurological problems	Grapp et al., 2012 (105)
Kl	Decrease	Depletion is associated with aging; may inhibit macrophage activation	Zhu et al., 2018 (106)
Ace	Decrease	Part of the renin-angiotensin system (RAS); increased activation may lead to increased blood pressure	Nakagawa et al., 2017 (58)
Cldn2	Decrease	Encodes for a water and cation channel	Steinemann et al., 2016 (107)
Steap1	Decrease	Overexpressed by glioblastoma cells	Moreaux et al., 2012 (108)
Cdkn1a	Decrease	Cell cycle inhibitor; reduces proliferation of NSCs	Kippin et al., 2005 (79)
Enpp2	Decrease	Potential function in oligodendrocyte precursor cells; increased expression following traumatic brain injury	Savaskan et al., 2007 (109)
Trpv4	Decrease	Critical for calcium signalling from initiated from an endothelial cell factor that activates vascular smooth muscle cells to vasodilate	Earley et al., 2005 (110)
Kcnj13	Decrease	Encodes for a potassium channel (not activated by depolarization, i.e., inwardly rectifying); may play a role in homeostasis of neurons and glial cells.	Papanikolaou et al., 2019 (21)
F5	Decrease	Encodes for coagulation factor V; critical for blood clotting	De Luca et al., 2017 (111)
Rgs6	Decrease	Important for gamma-aminobutyric acid B signalling	Maity et al., 2012 (112)

Supplemental References

1. Maruyama K, et al. CTLA-2 Alpha Is a Potent Inhibitor of Angiogenesis in Murine Ocular Tissue. *Antioxidants (Basel)*. 2021;10(3):456.
2. Green DP, et al. A Mast-Cell-Specific Receptor Mediates Neurogenic Inflammation and Pain. *Neuron*. 2019;101(3):412-420.e3.
3. Liang Q, et al. CD93 negatively regulates astrogenesis in response to MMRN2 through the transcriptional repressor ZFP503 in the developing brain. *Proc Natl Acad Sci U S A*. 2020;117(17):9413-9422.
4. Griffiths MR, et al. CD93 regulates central nervous system inflammation in two mouse models of autoimmune encephalomyelitis. *Immunology*. 2018;155(3):346-355.
5. Maas SLN, et al. Glioblastoma hijacks microglial gene expression to support tumor growth. *J Neuroinflammation.* 2020;17(1):120.
6. Fedorova O, et al. Orphan receptor NR4A3 is a novel target of p53 that contributes to apoptosis. *Oncogene.* 2019;38(12):2108-2122.
7. Vaithianathan T, et al. Functional roles of complexin in neurotransmitter release at ribbon synapses of mouse retinal bipolar neurons. *J Neurosci.* 2015;35(9):4065-4070.
8. Mondal A, et al. Lipocalin 2 induces neuroinflammation and blood-brain barrier dysfunction through liver-brain axis in murine model of nonalcoholic steatohepatitis. *Journal of Neuorinflammation.* 2020;17(1):201.
9. López J, Martínez A. Cell and molecular biology of the multifunctional peptide, adrenomedullin. *Int Rev Cytol.* 2002;221:1-92.
10. Larrayoz IM, et al. Adrenomedullin Contributes to Age-Related Memory Loss in Mice and Is Elevated in Aging Human Brains. *Front Mol Neurosci*. 2017;10:384.

11. Hur J-Y, et al. The innate immunity protein IFITM3 modulates γ-secretase in Alzheimer's disease. *Nature.* 2020;586(7831):735-740.

12. Moore RC, et al. Doppel-induced cerebellar degeneration in transgenic mice. *Proc Natl Acad Sci U S A*. 2001;98(26):15288-15293.

13. Yao Y, et al. Reducing Jagged 1 and 2 levels prevents cerebral arteriovenous malformations in matrix Gla protein deficiency. *Proc Natl Acad Sci U S A.* 2013;110(47):19071-19076.

14. Khan S, et al. IGFBP2 Plays an Essential Role in Cognitive Development during Early Life. *Adv Sci (Weinh).* 2019;6(23):1901152.

15. Wu T-N, et al. Effects of GADL1 overexpression on cell migration and the associated morphological changes. *Sci Rep.* 2019;9(1):5298.

16. Zeldich E, et al. The neuroprotective effect of Klotho is mediated via regulation of members of the redox system. *J Biol Chem*. 2014;289(35):24700-24715.

17. Vanharanta S, et al. Loss of the multifunctional RNA-binding protein RBM47 as a source of selectable metastatic traits in breast cancer. *Elife.* 2014;3.

18. Mishra BB, et al. Expression and distribution of Toll-like receptors in the brain during murine neurocysticercosis. *J Neuroimmunol.* 2006;181(1-2):46-56.

19. Krishna K, Redies C. Expression of cadherin superfamily genes in brain vascular development. *J Cereb Blood Flow Metab.* 2009;29(2):224-229.

20. Steinfeld R, et al. Folate receptor alpha defect causes cerebral folate transport deficiency: a treatable neurodegenerative disorder associated with disturbed myelin metabolism. *Am J Hum Genet.* 2009;85(3):354-363.

21. Papanikolaou M, et al. Glial and neuronal expression of the Inward Rectifying Potassium Channel Kir7.1 in the adult mouse brain. *J Anat.* 2019;235(5):984-996.

22. Li R, et al. TSG-6 attenuates inflammation-induced brain injury via modulation of microglial polarization in SAH rats through the SOCS3/STAT3 pathway. *J Neuroinflammation.* 2018;15(1):231.

23. Sato Y, et al. Cartilage acidic protein-1B (LOTUS), an endogenous Nogo receptor antagonist for axon tract formation. *Science.* 2011;333(6043):769-773.

24. Zega K, et al. Dusp16 Deficiency Causes Congenital Obstructive Hydrocephalus and Brain Overgrowth by Expansion of the Neural Progenitor Pool. *Front Mol Neurosci*. 2017;10:372.

25. Lacroix A, et al. COX-2-Derived Prostaglandin E2 Produced by Pyramidal Neurons Contributes to Neurovascular Coupling in the Rodent Cerebral Cortex. *J Neurosci.* 2015;35(34):11791-11810.

26. Oliva CR, et al. IGFBP6 controls the expansion of chemoresistant glioblastoma through paracrine IGF2/IGF-1R signaling. *Cell Commun Signal.* 2018;16(1):61.

27. Sewduth RN, et al. The ubiquitin ligase PDZRN3 is required for vascular morphogenesis through Wnt/planar cell polarity signalling. *Nat Commun.* 2014;5:4832.

28. Mishra SK. A nociceptive signaling role for neuromedin B. *J Neurosci.* 2012;32(25):8686-8695.

29. Ruden JB, et al. Parvalbumin interneuron vulnerability and brain disorders. *Neuropsychopharmacology*. 2021;46(2):279-287.

30. Naka H, et al. Requirement for COUP-TFI and II in the temporal specification of neural stem cells in CNS development. *Nat Neurosci.* 2008;11(9):1014-1023.

31. McNulty SE, et al. Differential roles for Nr4a1 and Nr4a2 in object location vs. object recognition long-term memory. *Learn Mem.* 2012;19(12):588-592.

32. Li C, et al. CaBP1, a neuronal Ca2+ sensor protein, inhibits inositol trisphosphate receptors by clamping intersubunit interactions. *Proc Natl Acad Sci U S A.* 2013;110(21):8507-8512.

33. Dosemeci A, et al. FAM81A protein, a novel component of the postsynaptic density in adult brain. *Neurosci Lett.* 2019;699:122-126.

34. Edamatsu M, et al. Hapln4/Bral2 is a selective regulator for formation and transmission of GABAergic synapses between Purkinje and deep cerebellar nuclei neurons. *J Neurochem.* 2018;147(6):748-763.

35. Callender JA, Newton AC. Conventional protein kinase C in the brain: 40 years later. *Neuronal Signal*. 2017;1(2):NS20160005.

36. Schaefer N, et al. Glycine receptor mutants of the mouse: what are possible routes of inhibitory compensation? *Front Mol Neurosci.* 2012;5:98.

37. Li X, et al. Loss of AP-2delta reduces retinal ganglion cell numbers and axonal projections to the superior colliculus. *Mol Brain.* 2016;9(1):62.

38. Gontier G, et al. Blocking IGF Signaling in Adult Neurons Alleviates Alzheimer's Disease Pathology through Amyloid-β Clearance. *J Neurosci.* 2015;35(33):11500-11513.

39. Sevlever D, et al. Genetically-controlled Vesicle-Associated Membrane Protein 1 expression may contribute to Alzheimer's pathophysiology and susceptibility. *Mol Neurodegener.* 2015;10:18.

40. Rich CA, et al. Olfactory ensheathing cells abutting the embryonic olfactory bulb express Frzb, whose deletion disrupts olfactory axon targeting. *Glia.* 2018;66(12):2617-2631.

41. Cole TB, et al. Elimination of zinc from synaptic vesicles in the intact mouse brain by disruption of the ZnT3 gene. *Proc Natl Acad Sci U S A*. 1999;96(4):1716-1721.

42. Kim SY, et al. Deficiency of Fhl2 leads to delayed neuronal cell migration and premature astrocyte differentiation. *J Cell Sci.* 2019;132(6):jcs228940.

43. Chen F, et al. The transcription factor NeuroD2 coordinates synaptic innervation and cell intrinsic properties to control excitability of cortical pyramidal neurons. *J Physiol.* 2016;594(13):3729-3744.

44. Wojcik SM, et al. An essential role for vesicular glutamate transporter 1 (VGLUT1) in postnatal development and control of quantal size. *Proc Natl Acad Sci U S A.* 2004;101(18):7158-7163.

45. Armenteros T, et al. BMP and WNT signalling cooperate through LEF1 in the neuronal specification of adult hippocampal neural stem and progenitor cells. *Sci Rep.* 2018;8(1):9241.

46. Sanz-Morello B, et al. Complex IV subunit isoform COX6A2 protects fast-spiking interneurons from oxidative stress and supports their function. *EMBO J.* 2020;39(18):e105759.

47. Seigneur E, Südhof TC. Genetic Ablation of All Cerebellins Reveals Synapse Organizer Functions in Multiple Regions Throughout the Brain. *J Neurosci*. 2018;38(20):4774-4790.

48. Perez DM. α1-Adrenergic Receptors in Neurotransmission, Synaptic Plasticity, and Cognition. *Front Pharmacol.* 2020;11:581098.

49. Kuang X-L, et al. Spatio-temporal expression of a novel neuron-derived neurotrophic factor (NDNF) in mouse brains during development. *BMC Neurosci.* 2010;11:137.

50. Martinelli DC, et al. Expression of C1ql3 in Discrete Neuronal Populations Controls Efferent Synapse Numbers and Diverse Behaviors. *Neuron.* 2016;91(5):1034-1051.

51. Kessi M, et al. Intellectual Disability and Potassium Channelopathies: A Systematic Review. *Front Genet.* 2020;11:614.

52. Yong HJ, et al. The unique expression profile of FAM19A1 in the mouse brain and its association with hyperactivity, long-term memory and fear acquisition. *Sci Rep.* 2020;10(1):3969.

53. Devanna P, Vernes SC. A direct molecular link between the autism candidate gene RORa and the schizophrenia candidate MIR137. *Sci Rep.* 2014;4:3994.

54. Mackie DI, et al. RAMP3 determines rapid recycling of atypical chemokine receptor-3 for guided angiogenesis. *Proc Natl Acad Sci U S A*. 2019;116(48):24093-24099.

55. Okada T, Suzuki H. The Role of Tenascin-C in Tissue Injury and Repair After Stroke. *Front Immunol.* 2021;11:607587.

56. Egertová M, et al. Localization of N-acyl phosphatidylethanolamine phospholipase D (NAPE-PLD) expression in mouse brain: A new perspective on N-acylethanolamines as neural signaling molecules. *J Comp Neurol.* 2008;506(4):604-615.

57. Lüscher C, Huber KM. Group 1 mGluR-dependent synaptic long-term depression: mechanisms and implications for circuitry and disease. *Neuron.* 2010;65(4):445-459.

58. Nakagawa P, Sigmund CD. How Is the Brain Renin-Angiotensin System Regulated? *Hypertension*. 2017;70(1):10-18.

59. Traylor M, et al. Genetic variation in PLEKHG1 is associated with white matter hyperintensities (n = 11,226). *Neurology.* 2019;92(8):e749-e757.

60. Cioli C, et al. Differences in human cortical gene expression match the temporal properties of large-scale functional networks. *PLoS One.* 2014;9(12):e115913.

61. Stern S, et al. Mechanisms Underlying the Hyperexcitability of CA3 and Dentate Gyrus Hippocampal Neurons Derived From Patients With Bipolar Disorder. *Biol Psychiatry.* 2020;88(2):139-149.

62. Vogelgesang S, et al. Analysis of the Serotonergic System in a Mouse Model of Rett Syndrome Reveals Unusual Upregulation of Serotonin Receptor 5b. *Front Mol Neurosci.* 2017;10:61.

63. Tang Y, et al. Protein kinase C-delta inhibition protects blood-brain barrier from sepsis-induced vascular damage. *J Neuroinflammation.* 2018;15(1):309.

64. Tomar VS, et al. Serine/threonine/tyrosine-interacting-like protein 1 (STYXL1), a pseudo phosphatase, promotes oncogenesis in glioma. *Biochem Biophys Res Commun*. 2019;515(1):241-247.

65. Hugnot JP, et al. Kv8.1, a new neuronal potassium channel subunit with specific inhibitory properties towards Shab and Shaw channels. *EMBO J.* 1996;15(13):3322-3331.

66. Manganas LN, et al. BASP1 labels neural stem cells in the neurogenic niches of mammalian brain. *Sci Rep.* 2021;11(1):5546.

67. Morita K, et al. BMP signaling alters aquaporin-4 expression in the mouse cerebral cortex. *Sci Rep.* 2021;11(1):10540.

68. Bar-Shira A, et al. CHRNB3 c.-57A>G functional promoter change affects Parkinson's disease and smoking. *Neurobiol Aging.* 2014;35(9):2179.e1-6.

69. Ruhl DA, et al. Synaptotagmin 17 controls neurite outgrowth and synaptic physiology via distinct cellular pathways. *Nat Commun.* 2019;10(1):3532.

70. Wang Y, et al. Pharmacological Bypass of Cockayne Syndrome B Function in Neuronal Differentiation. *Cell Rep.* 2016;14(11):2554.

71. Chen LT, et al. A candidate target for G protein action in brain. *J Biol Chem*. 1999;274(38):26931-26938.

72. Berecki G, et al. Novel Missense CACNA1G Mutations Associated with Infantile-Onset Developmental and Epileptic Encephalopathy. *Int J Mol Sci*. 2020;21(17):E6333.

73. Foulquier S, et al. The role of receptor MAS in microglia-driven retinal vascular development. *Angiogenesis.* 2019;22(4):481-489.

74. Galbraith KK, Kengaku M. Multiple roles of the actin and microtubule-regulating formins in the developing brain. *Neurosci Res*. 2019;138:59-69.

75. Salas R, et al. Abnormal social behavior in nicotinic acetylcholine receptor β4 subunit-null mice. *Nicotine Tob Res.* 2013;15(5):983-986.

76. Zelikowsky M, et al. The Neuropeptide Tac2 Controls a Distributed Brain State Induced by Chronic Social Isolation Stress. *Cell.* 2018;173(5):1265-1279.e19.

77. Khodosevich K, et al. Connexin45 modulates the proliferation of transit-amplifying precursor cells in the mouse subventricular zone. *Proc Natl Acad Sci U S A*. 2012;109(49):20107-20112.

78. Bunk EC, et al. Prox1 Is Required for Oligodendrocyte Cell Identity in Adult Neural Stem Cells of the Subventricular Zone. *Stem Cells*. 2016;34(8):2115-2129.

79. Kippin TE, et al. p21 loss compromises the relative quiescence of forebrain stem cell proliferation leading to exhaustion of their proliferation capacity. *Genes Dev.* 2005;19(6):756-767.

80. Fujita Y, et al. Netrin-G1 Regulates Microglial Accumulation along Axons and Supports the Survival of Layer V Neurons in the Postnatal Mouse Brain. *Cell Rep.* 2020;31(4):107580.

81. Bem J, et al. Wnt/β-catenin signaling in brain development and mental disorders: keeping TCF7L2 in mind. *FEBS Lett.* 2019;593(13):1654-1674.

82. Rosin JM, et al. Mice lacking the transcription factor SHOX2 display impaired cerebellar development and deficits in motor coordination. *Dev Biol.* 2015;399(1):54-67.

83. Silkworth WT, et al. The neuron-specific formin Delphilin nucleates nonmuscle actin but does not enhance elongation. *Mol Biol Cell.* 2018;29(5):610-621.

84. Abdolmaleky HM, et al. Epigenetic dysregulation of HTR2A in the brain of patients with schizophrenia and bipolar disorder. *Schizophr Res*. 2011;129(2-3):183-190.

85. Bedogni F, et al. Tbr1 regulates regional and laminar identity of postmitotic neurons in developing neocortex. *Proc Natl Acad Sci U S A*. 2010;107(29):13129-13134.

86. Mondal B, et al. Integrative functional genomic analysis identifies epigenetically regulated fibromodulin as an essential gene for glioma cell migration. *Oncogene.* 2017;36(1):71-83.

87. Inanobe A, et al. Inward rectifier K+ channel Kir2.3 is localized at the postsynaptic membrane of excitatory synapses. *Am J Physiol Cell Physiol*. 2002;282(6):C1396-1403.

88. Lv X, et al. TBR2 coordinates neurogenesis expansion and precise microcircuit organization via Protocadherin 19 in the mammalian cortex. *Nat Commun.* 2019;10(1):3946.

89. Birgner C, et al. VGLUT2 in dopamine neurons is required for psychostimulant-induced behavioral activation. *Proc Natl Acad Sci U S A.* 2010;107(1):389-394.

90. Okano HJ, et al. The cytoplasmic Purkinje onconeural antigen cdr2 down-regulates c-Myc function: implications for neuronal and tumor cell survival. *Genes Dev.* 1999;13(16):2087-2097.

91. Schneider EH, et al. Modulation of behavior by the histaminergic system: lessons from H(1)R-and H(2)R-deficient mice. *Neurosci Biobehav Rev*. 2014;42:252-266.

92. Zhang Y, et al. The effect of sphingomyelin synthase 2 (SMS2) deficiency on the expression of drug transporters in mouse brain. *Biochem Pharmacol.* 2011;82(3):287-294.

93. Planques A, et al. OTX2 Signals from the Choroid Plexus to Regulate Adult Neurogenesis. *eNeuro.* 2019;6(2):ENEURO.0262-18.2019.

94. Patil MJ. Prolactin receptor in regulation of neuronal excitability and channels. *Channels (Austin).* 2014;8(3):193-202.

95. Deák F, et al. Novel Cellular Functions of Very Long Chain-Fatty Acids: Insight From ELOVL4 Mutations. *Front Cell Neurosci*. 2019;13:428.

96. Ying S-W, et al. Targeted deletion of Kcne2 impairs HCN channel function in mouse thalamocortical circuits. *PLoS One*. 2012;7(8):e42756.

97. Joy MT, et al. Sulf1 and Sulf2 expression in the nervous system and its role in limiting neurite outgrowth in vitro. *Exp Neurol.* 2015;263:150-160.

98. Li X, Buxbaum JN. Transthyretin and the brain re-visited: is neuronal synthesis of transthyretin protective in Alzheimer's disease? *Mol Neurodegener*. 2011;6:79.

99. Varma VR, et al. Alpha-2 macroglobulin in Alzheimer's disease: a marker of neuronal injury through the RCAN1 pathway. *Mol Psychiatry*. 2017;22(1):13-23.

100. Yutsudo N, et al. fosB-null mice display impaired adult hippocampal neurogenesis and spontaneous epilepsy with depressive behavior. *Neuropsychopharmacology.* 2013;38(5):895-906.

101. Wang Y, et al. Overexpression of Epsin 3 enhances migration and invasion of glioma cells by inducing epithelial-mesenchymal transition. *Oncol Rep*. 2018;40(5):3049-3059.

102. Sergouniotis PI, et al. Phenotypic variability in RDH5 retinopathy (Fundus Albipunctatus). *Ophthalmology.* 2011;118(8):1661-1670.

103. Badaut J, et al. Aquaporin and brain diseases. *Biochim Biophys Acta*. 2014;1840(5):1554-1565.

104. Griffon N. CLIC6, a member of the intracellular chloride channel family, interacts with dopamine D(2)-like receptors. *Brain Res Mol Brain Res.* 2003;117(1):47-57.

105. Grapp M, et al. Molecular characterization of folate receptor 1 mutations delineates cerebral folate transport deficiency. *Brain*. 2012;135(Pt 7):2022-2031.

106. Zhu L, et al. Klotho controls the brain-immune system interface in the choroid plexus. *Proc Natl Acad Sci U S A.* 2018;115(48):E11388-E11396.

107. Steinemann A, et al. Claudin-1, -2 and -3 Are Selectively Expressed in the Epithelia of the Choroid Plexus of the Mouse from Early Development and into Adulthood While Claudin-5 is Restricted to Endothelial Cells. *Front Neuroanat.* 2016;10:16.

108. Moreaux J, et al. STEAP1 is overexpressed in cancers: a promising therapeutic target. *Biochem Biophys Res Commun*. 2012;429(3-4):148-155.

109. Savaskan NE, et al. Autotaxin (NPP-2) in the brain: cell type-specific expression and regulation during development and after neurotrauma. *Cell Mol Life Sci.* 2007;64(2):230-243.

110. Earley S, et al. TRPV4 forms a novel Ca2+ signaling complex with ryanodine receptors and BKCa channels. *Circ Res.* 2005;97(12):1270-1279.

111. De Luca C, et al. Neuro-Coagulopathy: Blood Coagulation Factors in Central Nervous System Diseases. *Int J Mol Sci.* 2017;18(10):E2128.

112. Maity B, et al. Regulator of G protein signaling 6 (RGS6) protein ensures coordination of motor movement by modulating GABAB receptor signaling. *J Biol Chem.* 2012;287(7):4972-4981.
